# Typological analysis of public-private partnerships in the veterinary domain

**DOI:** 10.1371/journal.pone.0224079

**Published:** 2019-10-31

**Authors:** Margot Galière, Marisa Peyre, Facundo Muñoz, Mariline Poupaud, Alain Dehove, François Roger, Isabelle Dieuzy-Labaye

**Affiliations:** 1 World Organisation for Animal Health (OIE), Paris, France; 2 CIRAD, UMR ASTRE, Montpellier, France, ASTRE, CIRAD, INRA, Univ Montpellier, Montpellier, France; University of Lincoln, UNITED KINGDOM

## Abstract

Public-Private Partnerships (PPPs) are defined as a collaborative approach in which the public and private sector share resources, responsibilities and risks to achieve common objectives and mutual benefits in a sustainable manner. PPPs are identified as a key solution to reinforce Veterinary Services. However only limited information is available on the scope, added value and enabling factors of PPPs in this sector. The aims of this study were to develop a typology of PPPs in the veterinary field and to identify key success factors and obstacles to their implementation. A structured questionnaire was sent to all 181 World Organisation for Animal Health (OIE) Member Countries and to 47 private contacts. 36 different variables characterizing PPP initiatives were collected. 97 examples of PPPs were retrieved from 76 countries. Dimensionality reduction techniques were combined with clustering and discrimination methods to establish a typology of PPPs and to derive a set of simple rules to classify new instances of PPPs. Three clusters were identified, separated according to two main variables: the type of private partners and the type of interaction. Cluster 1, transactional PPPs, represented the traditional understanding of PPPs by Veterinary Services, initiated and funded by the public sector, giving service delivery accreditation to mostly private veterinarians; cluster 2, collaborative PPPs, included partnerships between producer associations and public Veterinary Services, driven by trade interests; cluster 3, transformational PPPs, represented joint programs initiated and funded by private companies and initially driven by business development objectives. Specific success factors and key obstacles affecting the performances and sustainability of these initiatives were identified for each cluster. This study represents the first practical attempt to develop a meaningful typology of PPPs in the field of animal health and to identify fundamental obstacles currently inhibiting the development of PPPs, and suggests ways to support national Veterinary Services in overcoming these obstacles.

## Introduction

Public-Private Partnerships (or PPPs) are broadly defined as mutually beneficial collaborations between the public sector and a number of potential private collaborators [1]. Often considered in terms of large-scale collaborations between states, large national or transnational companies, and philanthropic organizations, PPPs nevertheless reflect a wide range of realities [1,2]. In the field of public health, PPPs correspond to new models of cooperation between states and companies, and this model is developed and disseminated within the United Nations [3–6]. Different categorization approaches have been developed for PPPs in public health, based for example on the distribution of ownership and risk bearing between the public and private sectors [7]. More recently, a “goal-oriented” categorization of PPPs in agribusiness has been developed by the Food and Agriculture Organization of the United Nations [8].

PPPs adapted to the field of veterinary services are defined as follows: “A Public-Private Partnership (PPP) is defined as a collaborative approach in which the public and private sector share resources, responsibilities and risks to achieve common objectives and mutual benefits in the field of veterinary services in a sustainable manner” [9]. PPPs are also identified as a key solution to reinforce Veterinary Services, defined as “governmental and non-governmental organizations that implement animal health and welfare measures, including private actors that are normally accredited or approved by the Veterinary Authority to deliver the delegated functions” [9]. The importance of PPPs in Veterinary Services is further stressed in the OIE Performance of Veterinary Services (PVS) pathway diagram on its website [10]. It is essential that these initiatives deliver clear guidance and support to national Veterinary Services in Member Countries towards promoting sustainable PPPs in the field of veterinary services.

However, only a limited number of PPP examples in the veterinary field have been fully described in the literature and there has been no attempt to define a typology of PPP initiatives in the fields of public and animal health based on field application [11–23]. Attempts to categorise PPPs identified from the literature review were based on theoretical considerations: a goal-oriented classification (most commonly used); based on the types of accountability; based on the geographical level; based on the level of engagement/responsibility/risk bearing [4,5,7,24–26]. To our knowledge, there has been no attempt to define a PPP typology based on a review of PPP initiatives in place in the fields of animal health or public health. Ajuha (2004) refers to a diversity of PPPs linking the state to professional organizations, civil society organizations and paraprofessionals or community relays to address the delivery of animal health services. This analysis highlights the difficulties encountered in implementing these approaches in the field, showing the need for further conceptualization efforts around the issue of their design [27]. PPPs in the veterinary domain have be referred in the literature as a partnership between the public veterinary authorities and multiple types of actors in the private sector, including private veterinary practitioners, Veterinary Statutory Bodies and private companies from the pharmaceutical or food industries, private diagnostic laboratories, and veterinary paraprofessionals (VPPs), farmers’ associations, producers’ associations, and community animal health workers, which increases the complexity of their categorization [19,21,28]. Moreover, a wide range of elements have been identified in the literature that impact the effectiveness and proper implementation of PPPs in different domains. However, it is essential to assess or confirm which elements (enabling factors or obstacles) are key in the veterinary domain in order to develop best practices for PPPs in this domain.

This work was done in the framework of the “Public Private Progress” initiative led by the OIE, with the support of the Bill & Melinda Gates Foundation and the collaboration of the French Research Institute for Agricultural Development (CIRAD). The objective of this three-year initiative (Nov. 2016–2019) is to support OIE Member Countries, particularly in Africa and Asia, to develop, as and when appropriate, sustainable PPPs to improve the quality of veterinary services and, consequently, animal health and the health and well-being of human populations, in line with one of the OIE’s three strategic priorities, namely strengthening Veterinary Services.

Given the wide range of PPP typologies available in the literature and the limited descriptive analysis of PPP examples in the veterinary domain, it is necessary to further explore these aspects in order to provide relevant recommendations on PPP best practices.

The aim of this study was to identify and characterize the different PPP initiatives existing worldwide in the veterinary domain in order to develop a categorization approach and to propose a typology adapted to this specific domain.

## Material and methods

### Data collection process

A structured questionnaire with open and closed questions was developed to collect data on current PPP initiatives worldwide and was pilot tested with six African OIE Member Countries, prior to developing the online version of the validated questionnaire using Survey Monkey software.

The type of data collected included: i) general information about the respondents, especially their affiliation by sector (public or private); ii) country-level information on two PPP initiatives supporting Veterinary Services perceived as successful by the respondent, including a brief description of the initiative, the partners involved, the period of implementation, the type of interaction between the partners, the funding mechanism, the governance mechanism, the type of activities implemented, impact assessment data if relevant, as well as general information about the strengths and weaknesses of reported PPPs; and iii) information on what the respondent perceived would ensure or impede good PPP implementation. The survey also captured respondents’ expectations about this study in terms of capacity building and any other related activities to promote an environment conducive to establishing PPPs, along with their expectations regarding feedback from this OIE survey. The questionnaire was written in French, Spanish and English and is available in the supplementary material (S1 File). It was pilot tested with 10 OIE regional representatives and CIRAD researchers before being used, to validate the relevance of its format and the adaptability of the questions to the different geographic contexts and PPP types. Respondents were selected using non-random convenience sampling targeting all OIE delegates worldwide. There was no a priori selection of PPP types or country location, and all the different types of PPPs described by the respondents were included in the analysis. The online questionnaire was sent by the OIE Director General (DG) to all OIE delegates of the 181 (at the time) Member Countries, mostly Chief Veterinary Officers, on 4 September 2017. The DG wrote a covering letter in the mail including the contact name and address of the researcher, the aims of the study and what would happen to the information provided. A reminder was sent by the DG a few days after the first response deadline of September 28. A two-week extension was given to the survey participants to respond. The online survey was closed on 8 December, i.e. three months after its launch.

A non-random snowball sampling approach was subsequently applied: the survey was sent to private contacts directly mentioned by the public respondents initially targeted. Private participants were also identified through personal contacts from OIE regional and sub-regional representatives and staff and study investigators. The objective was to collect views from the private sector to get a balanced assessment of PPPs in the field of veterinary services. No follow-up was conducted for private contacts obtained from the OIE delegate questionnaires in the following cases:

- No PPP example provided by the OIE delegate (or his/her representative) (n = 18)
- PPP example with no private contact provided, or no specific name associated with the private institution, or when the PPP involved multiple private contacts reported as a consortium (n = 7)
- PPP closely linked with the sanitary mandate with private partners being individual veterinary practitioners (n = 8)
- PPP focusing on the pet sector, whereas the survey focused on the livestock sector (n = 1)

### Data management and analysis

All the data were entered into an MS Excel spreadsheet database specifically developed for the survey. Manual text analysis techniques were used on open question answers to extract the most relevant keywords and to create new categorical variables (Table 1). The “Type of interaction” variable was split into two variables: 1) “Type of interaction perceived” and 2) “Type of interaction defined by the analysis”, as it became clear from the analysis that the type of interaction perceived by the respondents did not always match the nature and modalities of the PPP described. In the same way, the “Governance mechanism” variable was re-classified if the answer did not match the PPP description, or if misunderstanding of the question was obvious. The category “Not clear” was added if it was impossible to identify the appropriate mechanism from the description provided. A total of 36 variables were used to characterize the PPPs (S1 Table).

**Table 1:** Categorical variable derived from the text analysis of participant answers to open questions.

Variable	Categories		Comments
**General objective**	Animal infectious diseases, animal welfare, food safety, trade, antimicrobial resistance (AMR), veterinary education, veterinary legislation, multiple, and others (pets and conflict resolution).		These nine categories were defined based on the participants’ answers and harmonized according to the OIE’s PVS tools and its specification of the different fields of intervention for Veterinary Services.
**Main modality**	This variable was reclassified into 24 categories of modalities identified throughout all PPPs. Modalities used by the partnerships were reclassified into five categories based on the OIE Terrestrial Animal Health Code (Volume I, Sections 1, 3, 4, 5 and 6) and a category “Others”, in order to facilitate interpretation.		
**Intended duration**	Long term, fixed term and emergency situation (long-term PPPs that were only activated in case of emergency, such as disease outbreak control)	This variable was extracted from answers to the questions “current implementation state?” (past/ongoing/prospective) and “period of implementation?” (open question)	
**Resources**	Public, private, both, none	This variable was extracted from answers to the questions “public funding mechanism?” (yes/no) and “private funding mechanism?” (yes/no)	
**Type of public partner**	National and regional	This variable was extracted from answers to the questions “name of partners”	
**Type of private partner**	Private veterinarians/Veterinary Statutory Body (VSB)/veterinary association, producer organization/producers, private company, NGO/private foundation, para-public agency, consortium and others (individuals)	This variable was extracted from answers to the questions “name of partners”	
**Additional international partner**	Public (e.g. U.S. Agency for International Development (USAID) or Department for International Development (DfID), Non-Governmental Organizations (NGOs) /private foundation, public + NGO/private foundation, none		
**Type of interaction**	Communication, Consultation, Accreditation-Authorization-Delegation, Participation in joint programs.	Multiple choice answers were allowed for this variable, based on the four types of interactions defined in the OIE PVS tool [10]. If multiple types of interactions were mentioned, in order to ease the analysis, the type of interaction was reclassified into one category based on the inclusiveness or not of the different types of interactions.	

### Typology analysis

Several complementary methods from three different classes were used to establish a typology of PPPs and to derive a set of simple rules to classify new instances of PPPs (Table 2).

**Table 2:** Methods of multivariate data analysis that were combined to produce a typology of PPPs and classification rules.

Class	Methods	Objectives
Dimensionality reduction	Multiple Correspondence Analysis (MCA), Non-metric Multidimensional Scaling (MDS)	Visualize patterns, identify groups, Euclidean representation
Clustering	K-means, Agglomerative Hierarchical Clustering (AHC)	Classify PPPs
Discrimination	Classification and Regression Tree (CART)	Determine classification rules

To ensure that the derived typology reflects real structures in the data and is not an artifact of the chosen methods, we verified the consistency of the classification yielded by different combinations of methods from each family.

Dimensionality reduction methods were used to represent the PPPs in a low-dimensional Euclidean space of dimension d = 5. They made it possible to capture the main patterns of similarity among PPPs and to visualize these patterns and relationships. In particular, they revealed the clustered nature of PPPs and the number of such groups. Furthermore, they yielded a representation of PPPs in a Euclidean space that was used in turn by the clustering methods. Specifically, Multiple Correspondence Analysis (MCA) [29,30] and Non-metric Multidimensional Scaling (MDS) [31] were used.

K-means clustering [32] and Agglomerative Hierarchical Clustering (AHC) [31] were used to find the optimal classification of the PPPs in a specified number of clusters.

Finally, the Classification and Regression Tree method (CART) [33] was used to derive a simple binary Classification Tree, based on a few of the most discriminant variables, that matches the previously identified typology as closely as possible.

In order to validate both the consistency of the typology and the accuracy of the Classification Tree, the typologies derived from K-means clustering from both MCA and MDS representations, from HC and from CT, were compared.

These analyses were performed in *R* (R Core Team, 2018), using packages *FactoMineR* [34] for MCA, *MASS* [29] for MDS, *cluster* [35] for computing Gower’s dissimilarities, *stats* (R Core Team 2018) for K-means; *factoextra* [36] for AHC, and *rpart* [37] for CART. More information about data analysis is available in S2 File.

### Ethics statement

This work has been approved for implementation by the OIE and has not been reviewed by a specific ethical committee. No personal information was retrieved from the participants of the online study. The database has been double coded to ensure the anonymity of participants’ responses. This work and the reporting of this work do not present any potential risks to individuals or to the individual privacy of the study participants.

## Results

### Descriptive analysis of the data collected

#### Descriptive analysis of the respondents

**Fig 1:**
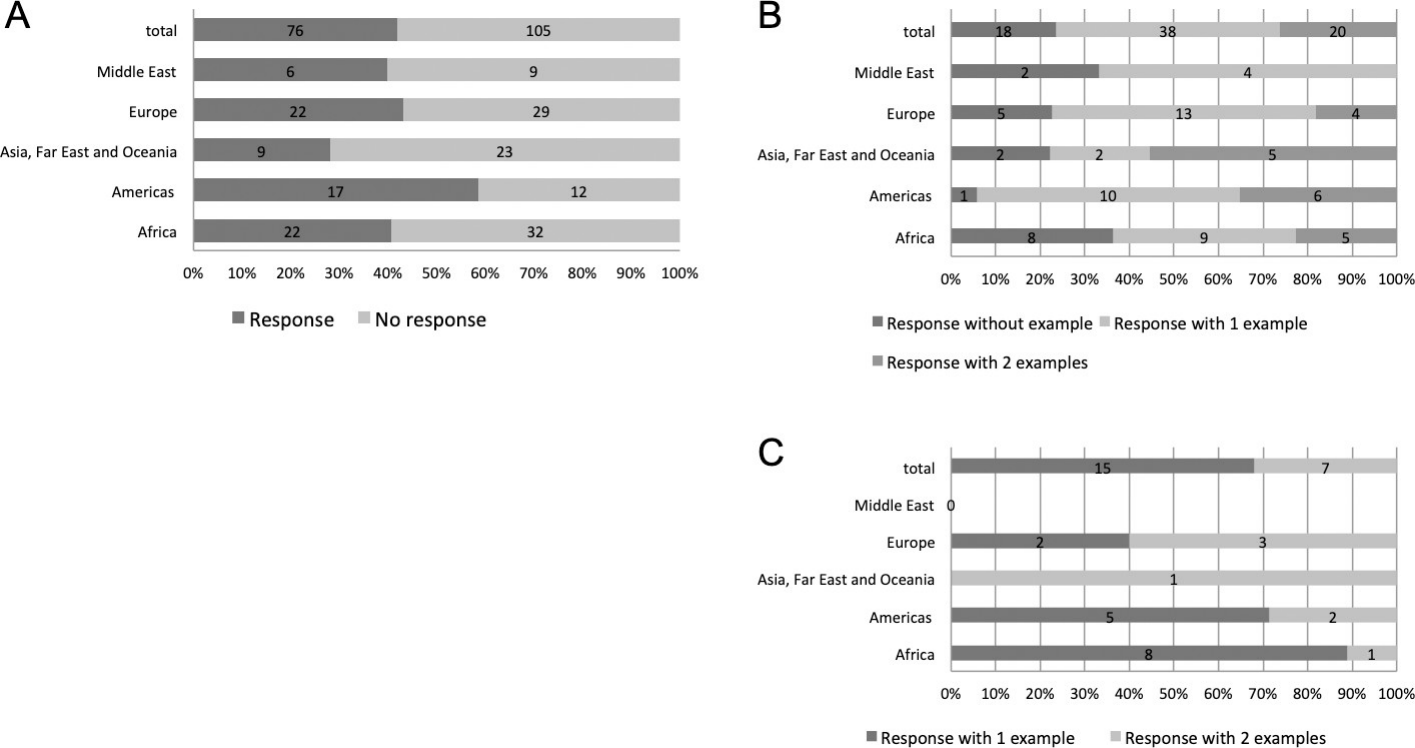
Distribution of the response rate and number of PPP examples according to OIE regions and respondent sectors. (A and B) Response rate from public sector respondents (OIE delegates); (C) response rate from private sector respondents.

The online questionnaire was sent to all OIE delegates in the 181 Member Countries, and responses were received from 76 countries (the global response rate was 42%), providing 81 different examples of PPP initiatives that strengthen veterinary services worldwide (Fig 1). The questionnaire was also sent to 47 private contacts (14 identified by the public respondents in the online survey and 33 from OIE and CIRAD direct contacts) with a similar response rate (47%, 22 private responses received) providing 29 PPP examples from 18 countries, including 13 already described by public respondents. This analysis is based on those 97 examples of PPPs described in the veterinary domain worldwide.

Differences in response rates were observed between OIE regions. In the Asia and the Pacific region, a lower response rate was obtained (28%), whereas the Americas region significantly exceeded the mean response rate (59%) (Fig 1A). These differences may reflect varying degrees of interest in PPPs in general.

Of the 76 responding countries, 50% provided one PPP example, 26% provided two examples, and 24% did not provide any specific examples but provided general information on what they perceived as obstacles to PPPs and general opinions on strengths and weaknesses (Fig 1B and 1C).

#### General objectives of the PPPs

**Fig 2:**
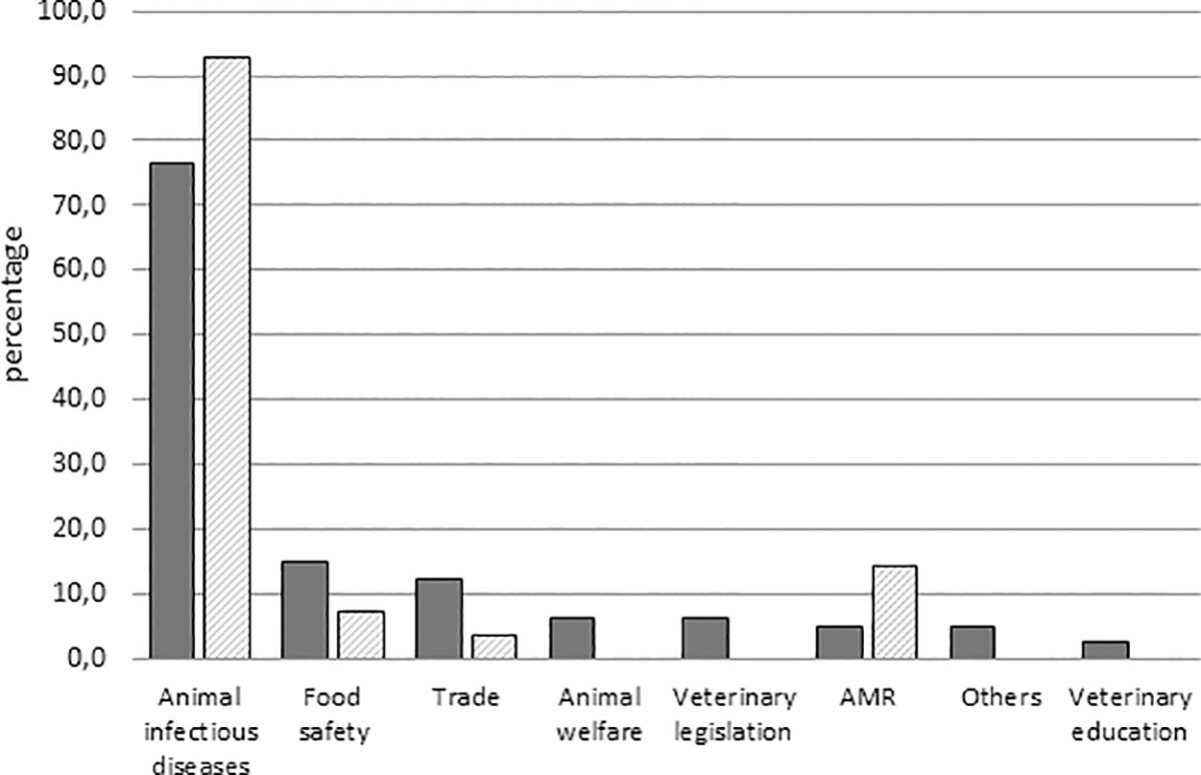
General PPP objectives as described by public respondents (solid bars) and private respondents (hatched bars).

83% of the PPPs reported by the public respondents had one main general objective, while 17% targeted multiple objectives (Fig 2). Most reported PPPs focused on animal infectious diseases (77% and 93% of the PPPs reported by public and private respondents respectively). This is consistent with the core mission of Veterinary Services, which aim to manage infectious diseases to protect the whole animal production value chain. Among the PPPs targeting animal infectious diseases, 76% focused on disease prevention and control, 6% on disease eradication, and 2% specifically targeted outbreak control in an emergency situation. 16% of these PPPs had multiple objectives. Food safety (14%) and fostering trade (product exports or animal imports, for example) (12%) were the second most represented objectives. PPPs targeting animal welfare and AMR control were relatively limited. This lower representation of the food safety objective could be linked to the fact that, in most countries, food safety issues are under the responsibility of human health authorities. PPPs targeting animal welfare were relatively limited, as this issue might not yet be perceived as crucial by both parties, especially in developing countries. AMR control as an objective was represented more in the PPP initiatives described by the private sector, as this topic has only recently been identified as a public issue in the field on a worldwide basis, but represents an important challenge for the private sector.

#### Modalities implemented in the PPPs

**Fig 3:**
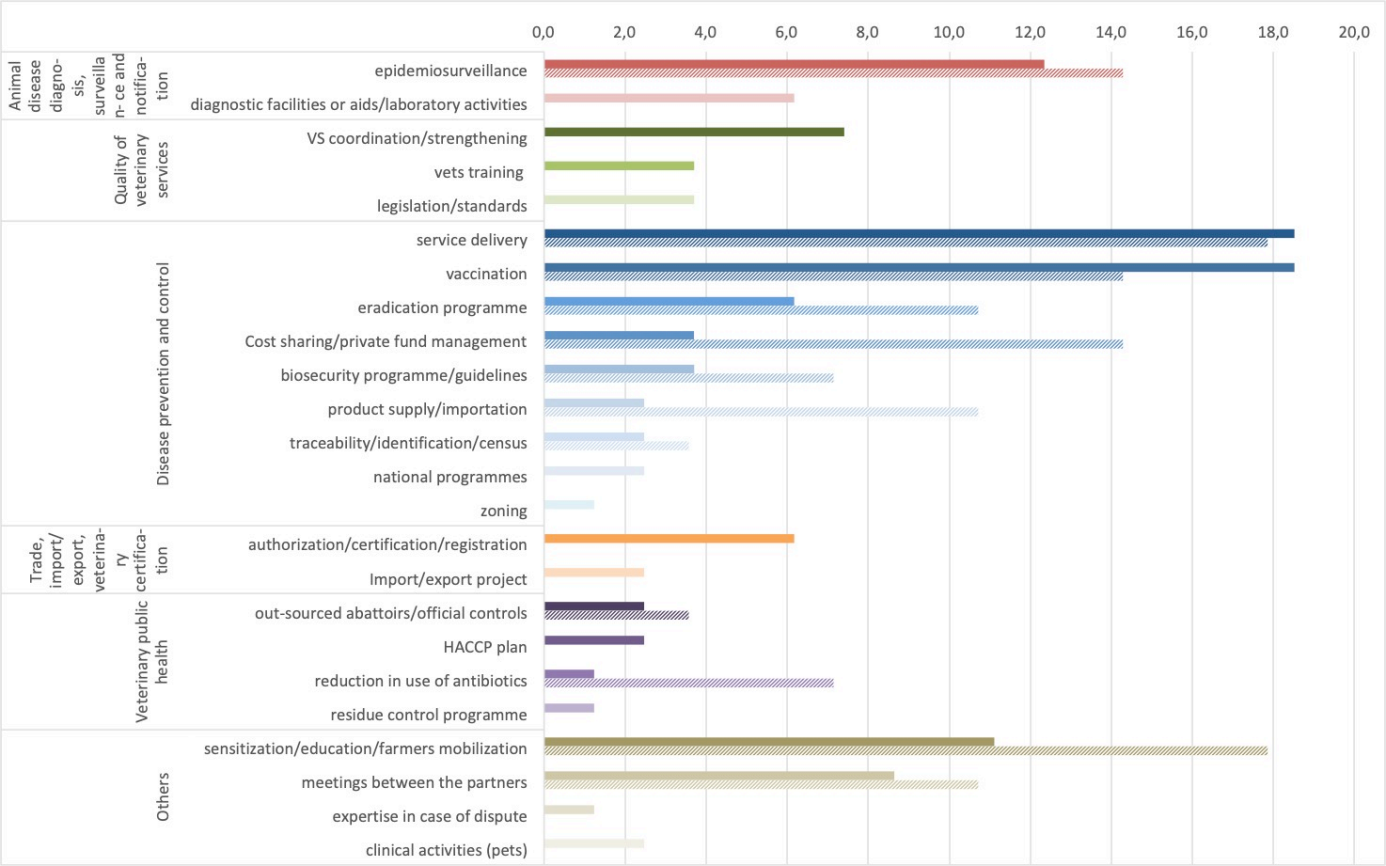
Modalities implemented under PPPs and reported by public respondents (solid bars) and private respondents (hatched bars).

Most PPPs described by public and private respondents related to activities on disease prevention and control (56%), education and communication (20%), diagnosis, surveillance and notification (18%), quality assurance (13%), trade (9%) and veterinary public health (7%) (Fig 3). Service delivery and vaccination were the two most represented modalities (19% each) (Fig 3). Service delivery included sanitary mandates, the installation of veterinary stations or mobile service delivery in remote areas. Service delivery modality was most often related to the implementation of vaccination campaigns, but not exclusively. Epidemiological surveillance was another important modality implemented in 12% of the PPPs described by public respondents. It included collaboration for passive and active epidemio-surveillance or the management of a surveillance platform. Public or farmer education and awareness raising or farmer mobilization represented an important mode of action (11%), as well as consultation through organized meetings between partners (9%). Such meetings included national commissions or national advisory councils targeting mainly animal infectious disease control or AMR.

The four most represented modalities described by the private sector were identical, with a strong emphasis on awareness raising/education/farmer mobilization (18%), service delivery (18%), vaccination (14%) and epidemio-surveillance (14%). The private sector respondents placed greater emphasis than public sector respondents on cost sharing, biosecurity programs, and product supply/importation, which is consistent with their greater relevance to the specific activities of the private sector.

It is interesting to note that this variable highlights some perception biases in the different points of view, with each respondent being more aware of and more inclined to talk about the modalities of interest to them. These data help to better understand the relative needs and focus of interest of each party, and will be of use when developing advocacy and guidelines for PPPs targeting both the public and the private sector.

#### Types of private partners engaged in the PPPs

Among the 81 PPPs described in this study, 78 mentioned public sector Veterinary Services as the public partner at the national level (96%), and only 3 at the regional or provincial level (4%). The types of private partners involved were more diverse. 75% of the PPPs described only one main private partner: private veterinarians, most often represented by the Veterinary Statutory Body (VSB) or a Veterinary Association (30%); producers, most often represented by producer organizations (associations or cooperatives) (23%), private companies (15%), para-public agency (4%), local NGO/private foundation (3%), others (such as private individuals) (2%); and 25% involved a consortia of private partners (an association of several partners collaborating on a project or program towards a common goal). Consortia partners included a producer organization (75%), private veterinarians (58%), private industrial producers from the livestock, avian, swine, meat or milk industries (37%) and, to a lesser extent, private companies (outside the livestock industry) (16%). Responses from the private sector showed a higher representation of private companies (31%) and producer organizations and private industrial producers (28%) in PPPs, and a lower representation of private veterinarians and VSBs (3%), simply reflecting the targeted sampling of private respondents.

Private companies mentioned in PPPs by public respondents were primarily involved in the supply and/or distribution of veterinary products, and were either global companies or local manufacturers and wholesalers. Other products included feed, disinfectants, breeding tools, pharmaceuticals and vaccines. Private companies were also mentioned in relation to support for field studies; providing seed and breeding stock; livestock exports; expertise to conduct veterinary inspections and technical support for customers.

**Fig 4:**
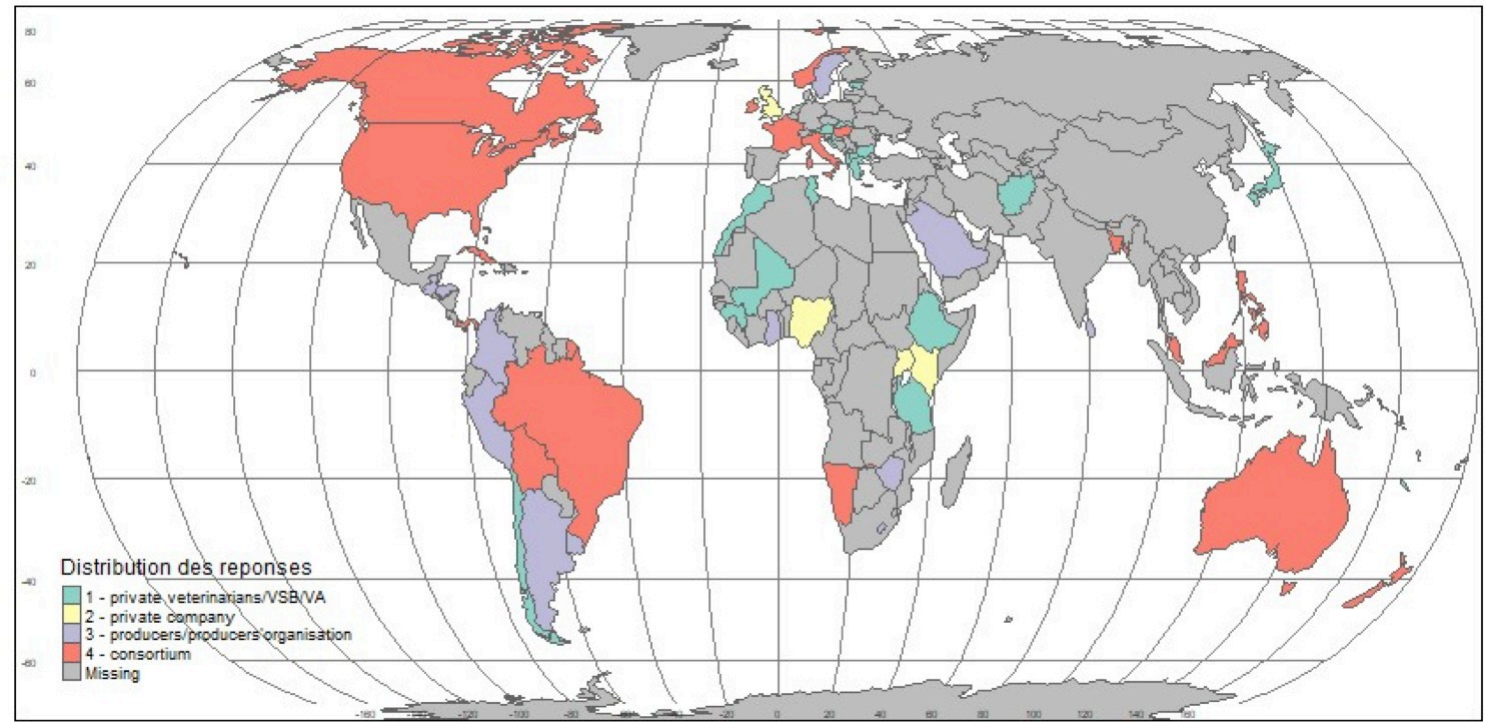
Geographic distribution of the different types of private partners involved in the PPPs described by public respondents.

A higher proportion of PPPs involving private veterinarians or VSBs were described in Europe and Africa, most often related to the sanitary mandate (Fig 4). PPPs involving private companies were more frequently described in Africa, often driven by development objectives, and to a lesser extent in Europe. A higher proportion of PPPs involving producer organizations were described in the Americas, particularly in Central and Latin America. Finally, consortia were mainly reported for PPPs in Europe, the Americas and Asia and the Pacific, whereas public respondents from Africa and the Middle East did not list any examples of PPPs involving consortia.

**Fig 5:**
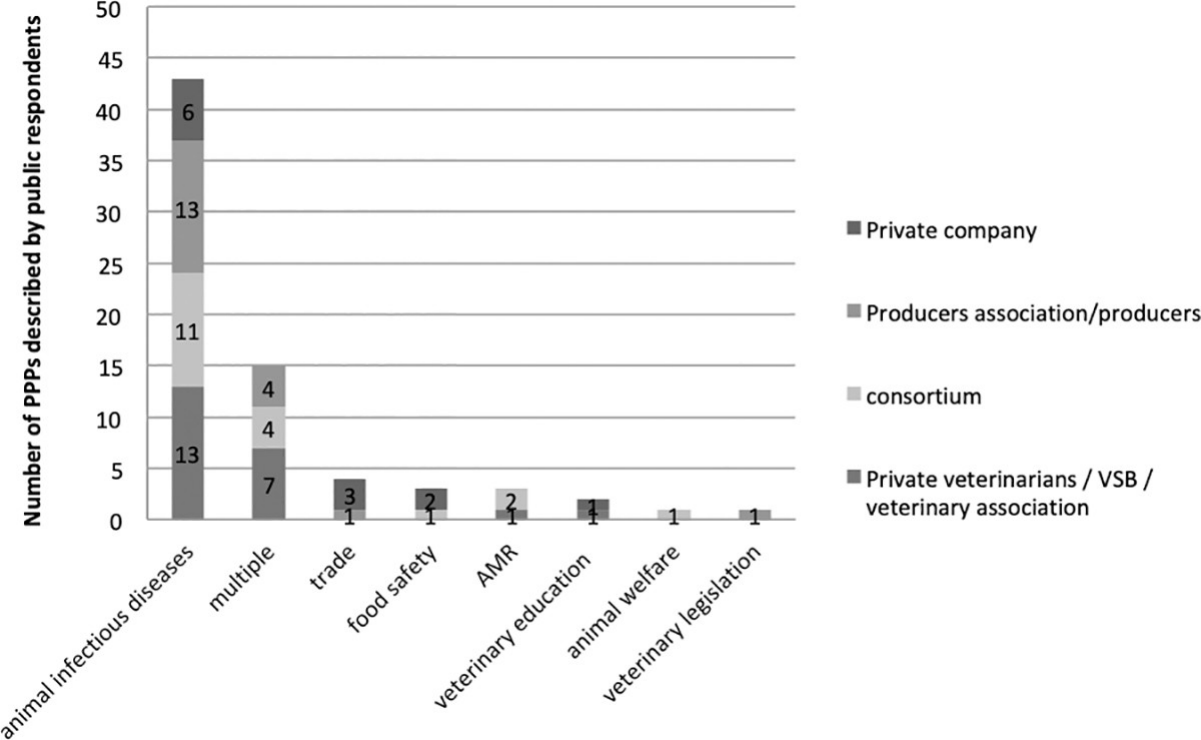
Distribution of the different types of private partners involved according to the PPP objectives, as described by public respondents.

All of the different types of private partners were involved in PPPs targeting infectious diseases. Private companies were predominant in PPPs focusing on trade, food safety and veterinary education objectives, but not in PPPs with multiple objectives (Fig 5). These multiple objective PPPs involved private veterinarians (most often linked to the sanitary mandate targeting animal infectious diseases as well as food safety and trade), consortia (collaborating on animal infectious diseases and other topics such as food safety, veterinary legislation, trade, AMR and animal welfare) and producer organizations (targeting animal infectious diseases and trade, food safety or veterinary legislation).

#### Who initiated the collaboration?

In 45% of the initiatives described, the partnership was initiated by both the public and the private partners, an indication of true public and private initiatives. When the partnership was initiated by just one of the sectors, public respondents mentioned public initiation in 37% of the cases; private respondents mentioned private initiation in 41% of the cases.

Collaborations involving a consortium or producer organizations were reported as being initiated by both the public and the private sectors. Collaborations involving private veterinarians, VSBs or veterinary associations were more often initiated by the public sector.

#### Types of interactions: A majority of joint programs

**Fig 6:**
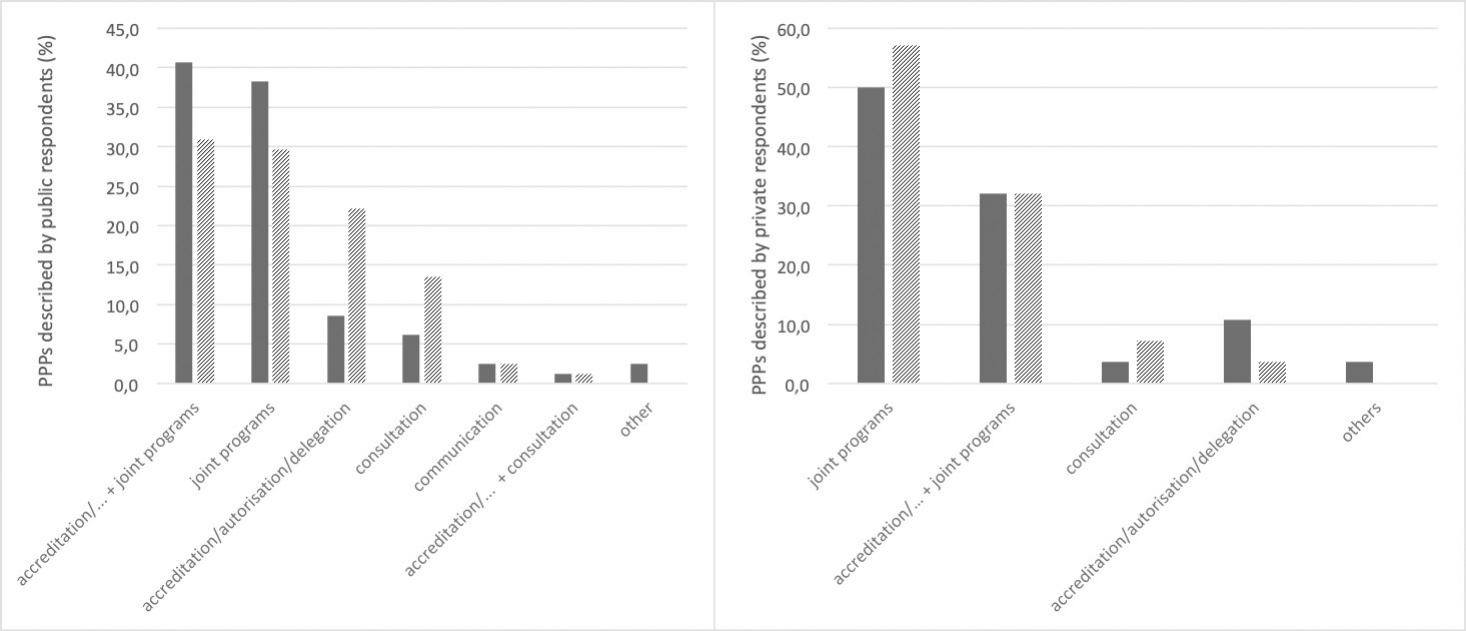
The different types of interactions reported in the PPPs. PPP interactions as described by (A) the public respondent and (B) private respondents (solid bars) and re-qualified by the analysis (hatched bars).

The majority of PPPs reported in this study by the public sector involved a high level of interaction between partners (even after re-qualification of the type of interaction) (Fig 6): 60% involved joint programming, reinforced by combination with accreditation, authorization or delegation in half of the cases, with an additional 22% linked to accreditation, authorization or delegation of activities to the private sector. Only 15% of the interactions related to consultation with interested parties, and 2.5% referred to communication from the public sector to the private sector.

31% of the perceived types of interaction in the PPPs reported by both the public and private sectors had to be re-qualified in the analysis (Fig 6). It is interesting to note that public respondents may tend to overestimate the level of PPP interactions, whereas private respondents tend to underestimate it (Fig 6).

The proportion of the “accreditation” type for PPPs described by the private sector was lower than for the public sector (Fig 6).

**Fig 7:**
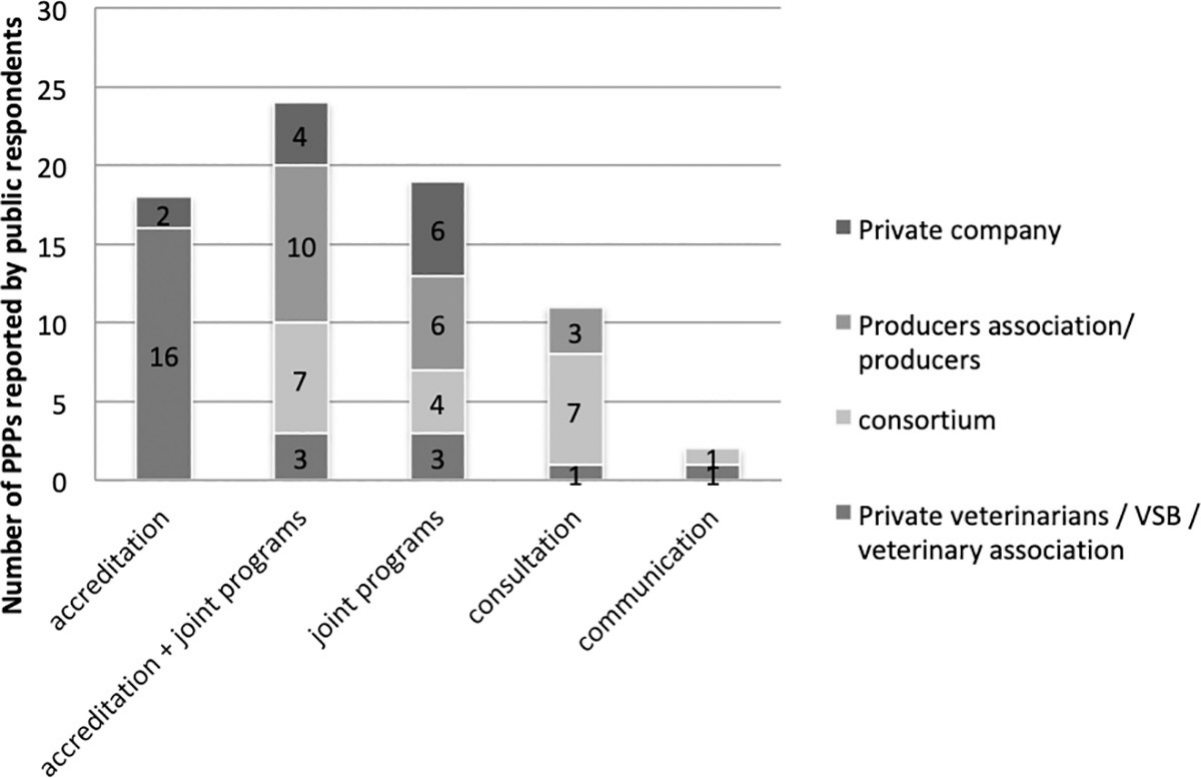
Types of main private partners involved in the PPPs reported by public respondents according to the different types of interactions.

Differences were observed in the types of private partners involved in the PPPs according to the type of interaction (Fig 7). Not surprisingly, PPPs involving private veterinarians, VSBs or veterinary associations were predominant in the accreditation/authorization/delegation category, as these reported partnerships most often relate to the sanitary mandate. Moreover, two PPPs in this category involved private companies: the first concerned the outsourcing of government abattoirs to the private sector in order to improve food hygiene and zoonosis surveillance; the second reported on the delivery of official veterinary tasks and controls by authorized private veterinarians employed by a private company. In contrast, consortia and producer associations dominated PPPs with interactions such as the combination of accreditation and joint programs or collaboration. In most PPPs involving private companies, the type of interaction was the joint program.

**Fig 8:**
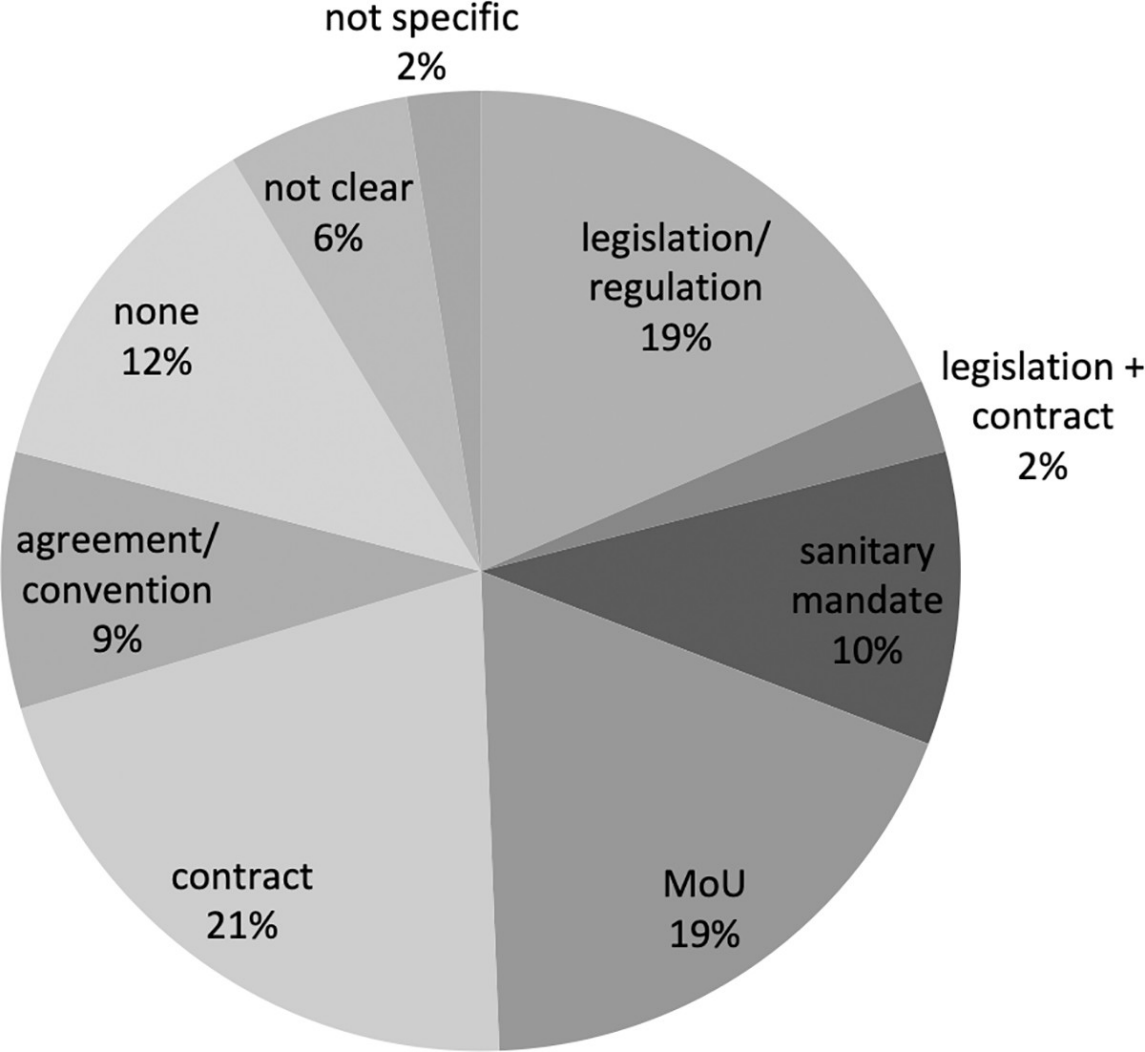
Distribution of different types of governance mechanisms reported by public respondents.

Differences were also observed in the modalities implemented in the PPPs according to the type of interaction (Fig 8): service delivery and epidemio-surveillance were mainly linked to accreditation (87% and 66% respectively), vaccination was mainly performed as part of joint programs with accreditation (20%) or without (53%), and meetings between partners were linked to consultation (defined here as collaboration, with the consulted party being involved in the subsequent decision, as opposed to simple communication) (86%).

#### Governance mechanisms

Governance mechanisms were classified according to their strength, ranging from PPPs governed by legislation to no specific governance mechanism: legislation/regulation > legislation + contract = sanitary mandate > memorandum of understanding (MoU) = contract > agreement/convention > others (none/not specific/not clear). A strong legislative basis could reflect more favorable conditions for sustainable PPPs. The sanitary mandate is a particular mechanism involving the delegation by the public sector of specific parts of veterinary service provision to private veterinarians, supported by legislation (mainly found in Europe and Africa (particularly West Africa). MoUs and contracts both reflect a formal written form of governance, with potential recourse to civil law, which it is not necessarily the case for an agreement/convention. 71% of PPPs reported by public respondents had a formal governance mechanism (legislation, sanitary mandate, MoU or contract), 9% were based on a weaker agreement/convention, and 12% were not based on any type of governance mechanism at all (Fig 8). Results from private respondents were similar to those obtained from public respondents, but sanitary mandates were not reported here because the questionnaire did not target any private veterinarians, VSBs or veterinary associations.

PPPs involving private veterinarians or a VSB were most often based on formal governance such as legislation, sanitary mandates or contracts (91%). Most PPPs involving consortia or producers were also based on a strong governance mechanism (59% and 69% respectively). All PPPs involving private companies had a formal governance mechanism in place, most often based on MoUs or contracts (75%).

#### Provision of resources

The PPPs reported by the public respondents were equally distributed in terms of the origin of resources between public (30%), private (27%) and both public/private (43%). Private respondents reported more jointly funded initiatives (51%) but fewer public-only funded initiatives (1%). Public funding only was linked to private veterinarians and sanitary mandates. Private funding only was linked to private companies, and both sources of funding to consortia or producer associations. The differences in the two groups could be explained by the fact that only public respondents described sanitary mandate PPPs, since no private veterinarians were surveyed.

#### Additional international partners

13 PPP initiatives described by public or private respondents involved an additional international partner (13%): 11 in Africa, one in the Middle East and one in Asia.

Three types of international partners were described: foreign public development agencies (such as USAID and DfID) or a United Nations organization (UNICEF, World Bank, FAO) (7%); NGOs from foreign countries (4%); a private foundation (BMGF) and a public development agency from a developed country (2%).

#### Intended duration

70% of the PPPs reported were considered as long_term, and 30% as fixed-term. There was no correlation observed between the type of partners involved and the intended duration of the PPP, although the distribution of long-term versus fixed-term was more prevalent for private veterinarians/VSBs/veterinary associations (79% versus 17%) than for private companies (67% versus 33%).

When resources were provided by the public sector, 86% of the PPPs were long-term initiatives, compared to 55% when the resources were provided by the private sector only. 78% of the initiatives were considered as long-term when both partners provided the resources. This suggests that the degree of public sector involvement in the provision of resources is an important factor in the sustainability of the initiatives.

50% of the initiatives involving an international partner were described as fixed-term, reflecting the fact that these development partners often operate in the initial stages of a project, with limited duration of funding and the goal of initiating locally-resourced follow-up work and collaborations.

#### Key success factors and obstacles of PPPs

**Fig 9:**
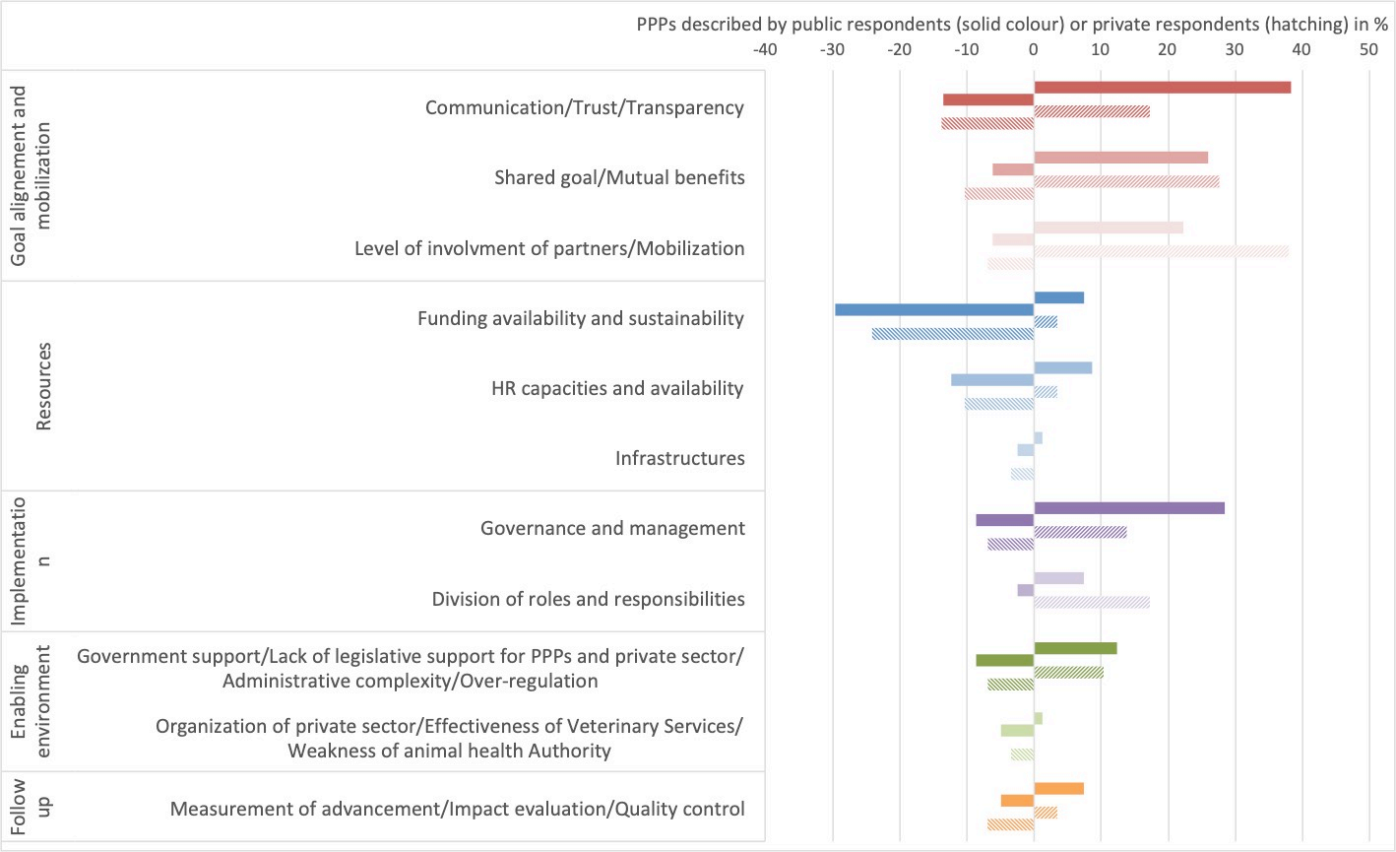
Key success factors and obstacles of PPPs. Key success factors (right side) and obstacles (left side) of PPPs reported by public respondents (solid bars) and private respondents (hatched bars) and classified in five different categories: goal alignment and mobilization, resources, implementation, enabling environment and follow-up.

While many respondents reported on the strengths and/or weaknesses of the actions and programs implemented in the course of their PPP, we focus in the following descriptive analysis on responses concerning key success factors (KSFs) and obstacles related to the partnership itself (Fig 9). Goal alignment and mobilization of partners was the most reported KSF category (21–38%) (Fig 9). It included communication between partners, trust and transparency, shared goals and mutual benefits, and the level of involvement of partners. KSFs linked to implementation were the second most important category (7–29%), with governance or management and a clear division of roles and responsibilities considered as important in the success of the partnership. Resources (2–8%), an enabling environment including government support (12%), and organization of the private sector (2%) were also reported as KSFs. Resources were most often reported as an obstacle, particularly the availability and sustainability of funding. The other reported obstacles were the lack of KSFs previously described (Fig 9).

Differences were observed between public and private responses, with the public sector more inclined to mention communication/trust/transparency and governance as KSFs (38% by public versus 18% by private), while the private sector seemed to insist more on the level of involvement of partners, their engagement (38% by private versus 21% by public), as well as the division of roles and responsibilities (17% by private versus 8% by public). However, these nuances must be treated with caution given the relatively smaller sample of private respondents in the survey.

**Fig 10:**
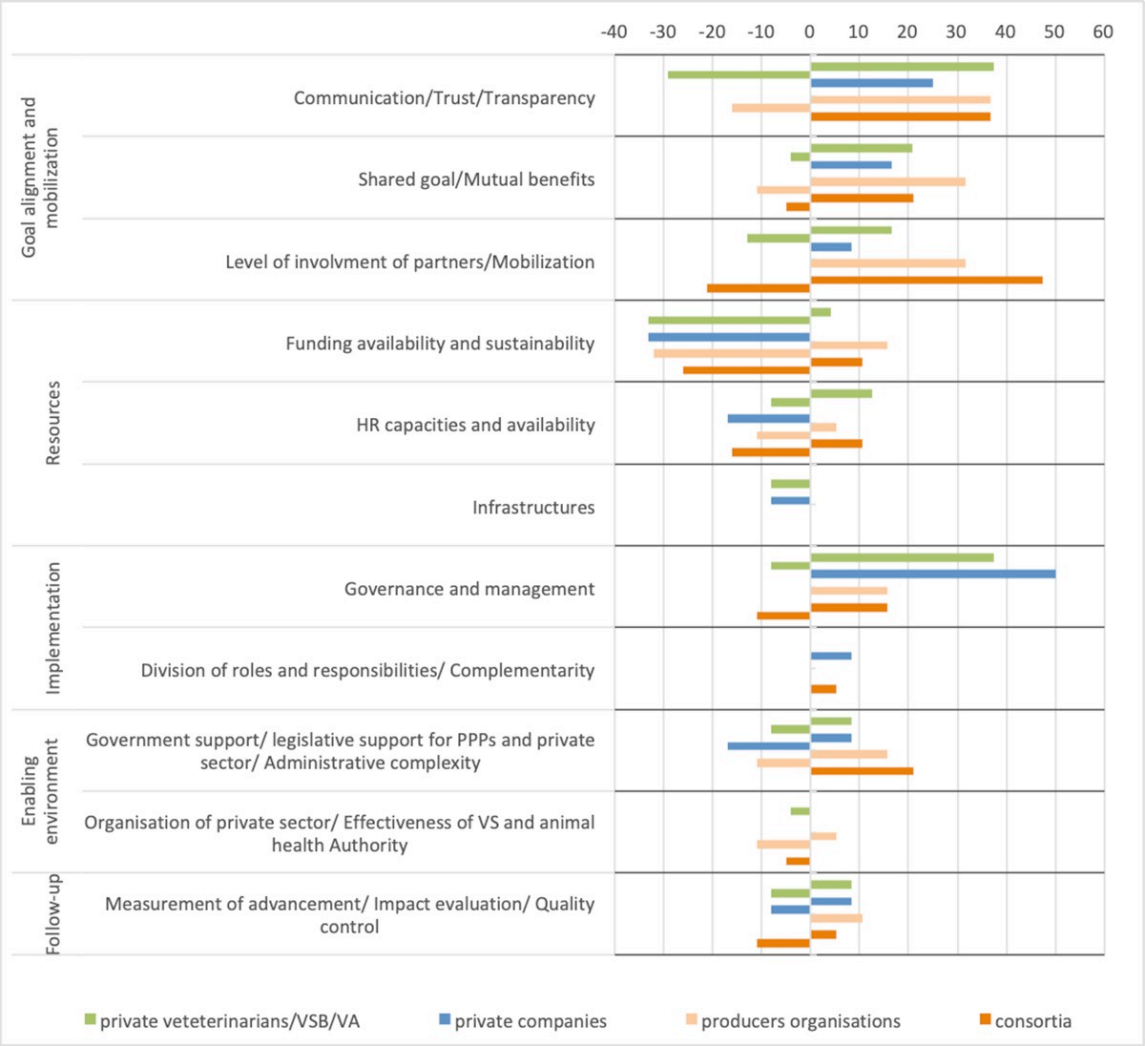
Key success factors and obstacles of PPPs reported by public respondents for the four major types of main private partners involved.

Looking at the distribution of the KSFs and obstacles according to the type of private partners involved in the PPPs, the study highlighted that communication and trust, and financial and human resources were the most commonly mentioned obstacles for PPPs involving private veterinarians, a VSB or a veterinary association (38% and 37%) (Fig 10). Those two categories, in addition to factors relating to the enabling environment (including the lack of private sector organization), were also important for PPPs involving producer associations. Governance was the most important KSF reported for PPPs involving private companies (40%). For these types of PPPs, obstacles were mainly funding availability and sustainability (35%), limited human resource capacities and availability (18%), and a lack of legislative support for PPPs and administrative complexity (18%). For PPPs involving a consortia, the level of involvement of partners (47%), communication/trust (38%) and government support (21%) were the most important KSFs; a lack of resources and governance were the most reported obstacles for these PPP initiatives.

### PPP typology analysis: Three distinct clusters

**Fig 11:**
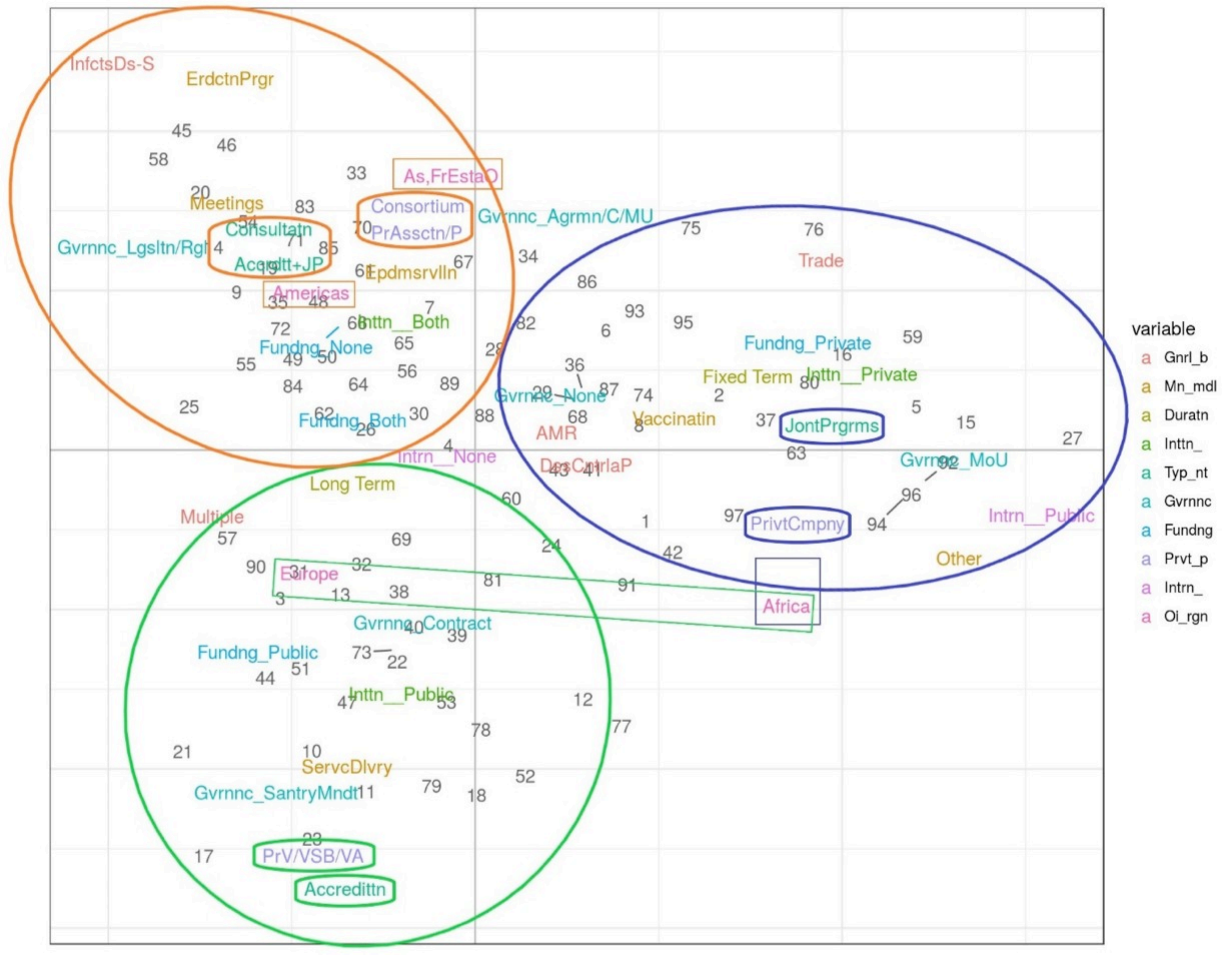
The three clusters of PPPs reported in the survey and analyzed by multiple correspondence analysis (MCA) and hierarchical clustering. The first two dimensions accounted for 20% of the PPP variance. The three clusters identified by MCA and K-means methods are displayed as circles including the PPP initiatives and the main variables characterizing the clusters are highlighted: cluster 1 (green circle and boxes); cluster 2 (orange circle and boxes); cluster 3 (blue circle and boxes).

The factorial analysis of data collected through the survey highlighted three distinct clusters of PPPs providing the basis for a PPP typology proposal (Fig 11 and Table 3). The three different methods used (MCA, MDS and hierarchical clustering) provided convergent outputs (S1 Fig). Two main variables were essential to explain the differences between the three clusters: the category of main private partner collaborating with the public sector and the type of interaction between the partners (S2 Fig). Other variable categories were identified to further characterize each of the three clusters: the main modality and governance categories had a greater contribution than the OIE region, initiation of the collaboration, objective, funding, intended duration and additional international partner categories (S3 Fig and Table 3). Cluster 1 (including 26% of the reported PPPs) involved private veterinarians, a VSB or a veterinary association as the main private partner(s) and was closely linked to accreditation as the governance mechanism (Fig 11, Table 3). Cluster 2 (including 40% of the PPPs) mainly involved consortia and producer associations and was closely linked to joint programs and collaboration (Fig 11, Table 3). Cluster 3 (including 34% of the PPPs) involved mainly private companies (local and multinational) and was closely linked to joint programs.

**Table 3:** Main variable categories associated with the three PPP clusters identified in the factorial analysis.

Variables	Cluster 1	Cluster 2	Cluster 3
**Main private partner**	Private veterinarians, VSB or veterinary association	Producer organization or consortium	Private company
**Type of interaction**	Accreditation	Accreditation + participation in joint programs or consultation	Participation in joint programs
**Main modality**	Service delivery	Eradication program, meetings between the partners or epidemio-surveillance	Vaccination
**Governance**	Sanitary mandate or, to a lesser extent, contract	Legislation or agreement/convention	MoU
**Main region**	Europe or Africa	Americas or Asia/Pacific	Africa
**Provision of resources**	Public sector	None or both the public and private sectors	Private sector
**Initiation of collaboration**	Public partner	Both the public and private partners	Private partner
**Objective**	Multiple (infectious diseases and food safety, trade, animal welfare, etc.)	Infectious diseases–several specific objectives (prevention, control, eradication, emergency outbreak control)	Infectious disease control and prevention, trade or AMR
**Intended duration**	Long term	Long term	Fixed term
**Additional international partner**	None	None	Additional international partners from public sector or foundation/NGO

## Discussion

This study documented 97 examples of PPPs implemented in the veterinary domain worldwide and categorized them into three main clusters, offering new insights and understanding of the types of PPPs currently in place in the veterinary domain. To our knowledge, this study represents the first attempt to develop a typology of PPPs in the veterinary domain, based on existing initiatives and the background scientific literature on this topic.

The general objectives of the PPPs identified in this study were mostly related to animal infectious diseases. Regarding modalities implemented under the PPPs, it is interesting to note that this variable highlights some perception biases in the different points of views with each respondent being more aware of and more inclined to talk about the modalities of interest to them. Concerning the actors who initiate the PPP, public and private partners were more likely to mention PPPs initiated by their own institution, which can explain the differences in responses from the two groups. This bias could also explain the limited number of similar PPPs described by both types of respondent for a given country (<50%). The fact that the proportion of “accreditation”, the highest level of interaction, described by the private sector was lower than for the public sector is probably linked to the private respondent sampling bias, as the survey was not sent to individual private practitioners, VSBs or veterinary associations. As expected, the veterinary sanitary mandate (involving private veterinarians, VSBs or veterinary associations), which reflects sub-contracting rather than a collaborative partnership, was highlighted mainly as a public initiative. This may reflect the need for Veterinary Services in many countries to adapt to reduced expenditures on public services. This could result from the structural adjustment policies initiated in the 1990s in many low- and middle-income countries (LMICs). To compensate for the subsequent cessation of many public services provided to farmers, such as vaccination, the private sector was involved and was tasked with conducting some of the traditional public activities.

The results on the intended duration of the PPP suggest that the degree of involvement of both the private and the public sector in the provision of resources is an important factor in the sustainability of the initiatives. This observation is also made by Delmotes *et al*. regarding PPPs in public health [38]. This output also reflects the fact that international development partners often operate in the initial stages of a project, with limited duration of funding and with the goal of initiating locally-resourced follow-up work and collaborations.

A majority of PPPs reported in this study were true PPPs initiated by both public and private partners. This common partnering initiative does not imply similar motivations for the two sectors. The specific interests of both partners may diverge, for example in cases where trade in and export of animal products are at stake, the private sector may seek to optimize its revenues and subsequent profits, while the public sector is looking to maximize exports and to source foreign exchange. This was clearly highlighted in the KSFs under the goal alignment and mobilization of partners category and the importance for the private sector of a clear division of roles and responsibilities. Our results show that as with PPPs in other domains, the specific interests of each type of partner should be acknowledged and understood [6,39].

This study provides a robust typological analysis based on the response rate, the number of PPP initiatives identified and included in the analysis (n = 97), and the convergence of outputs between the three methods used in the factorial analysis. The PPPs reported were considered as a representative view of the different types of PPPs implemented worldwide, even though some OIE regions were more represented than others. The response rate (45%) observed with the online survey is comparable to most surveys previously conducted by the OIE, and the 97 examples provided a good overview of the types of PPPs currently being implemented in the different regions of the world. The differences in response rate observed between the OIE regions may reflect varying degrees of interest in and awareness of PPPs; for example, the Americas’ particular interest in PPPs may be related to the strong presence of producer associations. Given that the private respondent sampling frame was not exhaustive, unlike the public one, it is difficult to draw general conclusions from the private respondents’ feedback. However, some general trends were highlighted by the analysis, especially when looking at the 13 PPPs described by both sectors. However, the range of private partners described in the survey reflected the definition of PPPs proposed in this study, and extended it far beyond the traditional understanding of PPPs by Veterinary Services, which tends to restrict private partners to only private veterinary practitioners and Veterinary Statutory Bodies [18,19,22].

**Fig 12:**
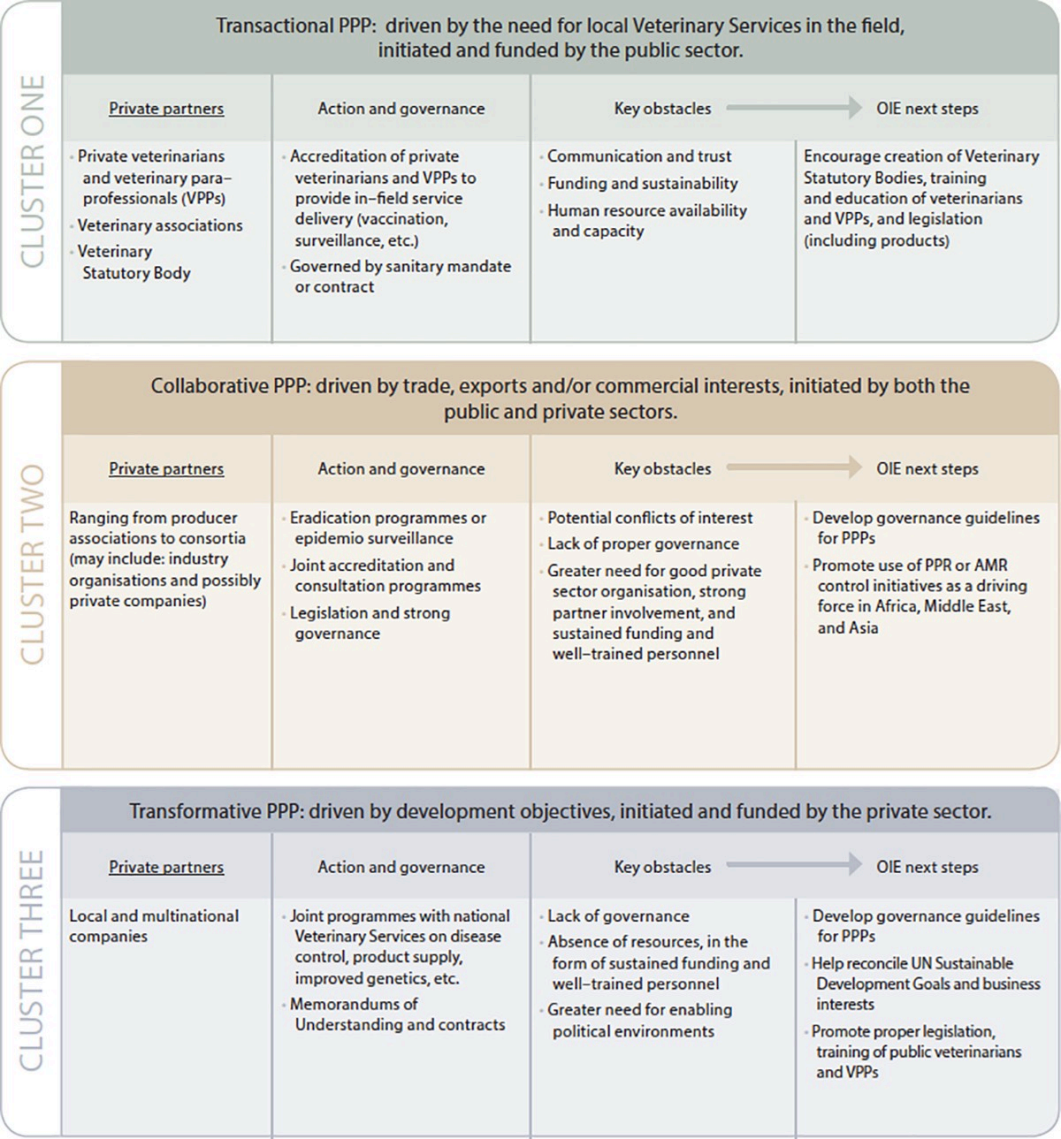
Typology of PPPs in the field of Veterinary Services.

Based on the outputs of this study, we propose a typology of PPPs in the field of Veterinary Services (Fig 12):

- Cluster 1, ***transactional PPPs***: the (national) procurement of discrete animal health/sanitary services from private veterinary service providers, usually veterinary businesses, veterinary para-professionals (VPPs) or associations. These are initiated and funded by the public sector, possibly with further payment from the producer that benefits from the service. The governance is a client (government)/private provider relationship. The private provider is contracted or given a sanitary mandate. A good partnership is essential to delivering optimal outcomes for both parties.
- Cluster 2, ***collaborative PPPs***: ajoint commitment between the public sector and end-beneficiaries, often producer associations, sometimes a consortium of producer associations and a range of other interested private organizations such as veterinary associations, to deliver mutually agreed (national) policies/outcomes. Collaborative PPPs are often driven by trade and possibly export interests, and are therefore often jointly initiated and funded, possibly with payment by commitment of resources other than money. Governance ranges from regulation through legislation (e.g. joint delivery programs, strong governance) to non-official agreements (e.g. consultation on animal health policies, weak governance), and decision-making is shared between the collaborating parties.
- Cluster 3, ***transformative PPPs***: establishing sustainable capacity to deliver otherwise unattainable major programs. Often initiated by the private sector but sanctioned by, and working with, the national Veterinary Services. Funded by large national or multinational private sector companies (possibly initially enabled by international aid, or the national/international philanthropic/charitable sector) to achieve long-term sustainable business returns and/or a public good commitment from the private partner. Joint governance, such as a MoU, with the public partner.

The output of this study made it possible to illustrate the three clusters with the representative examples given in Fig 12. However, the geographic distribution observed between the different types of PPP clusters (cluster 1 mostly reported in Europe and Western Africa; cluster 2 in the Americas and Asia/Pacific; and cluster 3 in Africa) should be taken with caution based on the representativeness of the study sample.

This categorization has been drawn from a rigorous typological analysis, based on practical examples of PPP implementation in the field, taking into consideration 36 different variables characterizing the PPPs (e.g. type of private partner; type of governance; type of interaction; objectives; modalities, region of implementation; provision of resources; intended duration; etc.) (S1 Table). The transactional PPP category represents a type of standard contracting between two parties; however, the use of a different terminology here distinguishes it from the other two categories, which also refer to some kind of contracting. This categorization, adapted to empirical data on PPPs in the veterinary domain, relates to the public health PPP typology proposed by Kraak *et al*., and to outputs from other studies on the empirical analysis of PPPs (not linked to the health domain) [40–42]. This categorization is more inclusive and allows for greater flexibility than the “goal-oriented” one recently proposed by FAO, for example partnerships that aim to develop agricultural value chains, partnerships for joint agricultural research, innovation and technology transfer, partnerships for building and upgrading market infrastructure, and partnerships for the delivery of business development services to farmers and small enterprises [8].

The main objective of this work was to better understand the different types and current status of PPPs in the field of veterinary services worldwide, to enable the OIE to draw recommendations and identify actions to be undertaken to support the emergence of more PPPs in order to strengthen National Veterinary Services if considered relevant in a given context. The analysis of the survey outputs provided critical elements on what to consider with a view to unleashing the potential for more PPPs in the field of Veterinary Services, as well as preliminary suggestions on what the OIE can implement to drive change in each cluster, if and when desirable. In their study, Buse & Walkman provide recommendations on how to improve WHO PPP strategy without accounting for their diversity and categorization [43]. Our study highlights the importance of making generic recommendations (when possible) based on PPP diversity in the veterinary domain to provide adapted and action-oriented guidelines that could be promoted by international organisations (such as the OIE). It is clear, however, that generic guidelines on PPP implementations may not be appropriate, but should be based on rigorous typological analysis of the initiatives in place to highlight the specific variables that would ensure success of the efforts made.

Resources and especially funding emerged as an important obstacle. However, it is interesting to note that even if this obstacle was overcome, ensuring sufficient and adapted resources would not be enough to guarantee success of the PPPs. Effective communication and trust between the partners along with proper governance mechanisms were shown to be key elements of this success. Moreover, the degree of public sector involvement in the provision of resources was shown as an important factor in establishing long-term initiatives, as demonstrated by the long-term versus fixed-term distribution of reported PPPs.

## Conclusions

This study provides an analysis of PPPs in the veterinary domain implemented around the world. 97 examples of PPP initiatives were described, illustrating the considerable need for PPPs to strengthen Veterinary Service activities worldwide and the importance of doing so. This study made it possible to develop the first typology of PPPs in the field of veterinary services, to identify fundamental obstacles currently inhibiting the development of the different types of PPPs and to support national Veterinary Services in overcoming these obstacles. PPPs in the veterinary domain can be categorized in three main groups (transactional, collaborative and transformational), mainly defined by the type of private partner engaged and the type of governance overarching the partnership. This work also highlighted the need to develop practical guidance taking into consideration the great diversity of PPPs in order to define the enabling environment needed for the initiation, implementation and maintenance of impactful and sustainable partnerships towards the strengthening of National Veterinary Services.

## Supporting information

S1 File Online questionnaire. (PDF)

S2 File Factorial analysis. (DOCX)

S1 Table List of variables used to characterize the PPPs. (DOCX)

S1 Fig Convergence of the outputs from the four factorial analysis methods implemented in this study. The three PPP (public-private partnership) clusters are clearly distinguished by the three methods: MCA = Multiple Correspondence Analysis; MDS = Multidimensional Scaling; CT = Classification Tree, HC = Hierarchical Clustering.
(TIF)

S2 Fig Cluster differentiations of the PPPs according to the three clustering approaches: the PPPs are grouped in three different clusters by the k-means method. (A); those clusters can be differentiated according to two main variables: the type of private partners and the governance mechanism, as shown with the hierarchical clustering and classification tree methods (B and C).
(TIF)

S3 Fig Correlation between variables and MCA dimensions (left side) and relative contribution of the 10 most influential categories (right side).
(TIF)
