# Supplementary material for: Typological analysis of public-private partnerships in the veterinary domain
Source: PLoS One. 2019 Oct 31;14(10):e0224079. doi: 10.1371/journal.pone.0224079 (PMC6822735; doi:10.1371/journal.pone.0224079)
Supplement: S1 File — (PDF) [file pone.0224079.s001.pdf]

WORLD ORGANISATION FOR ANIMAL HEALTH
Protecting animals, preserving our future

Public Private Partnership survey / Enquête Partenariats Public Privé / Encuesta Asociaciones Público-Privadas

1. Language / Langue / Idioma

* 1. Please choose your language / Merci de choisir votre langue / Por favor elija su idioma

- ◯ English
- ◯ Français
- ◯ Español

WORLD ORGANISATION FOR ANIMAL HEALTH
Protecting animals, preserving our future

2. Introduction to the Survey

During the 85th General Session of the World Assembly of the Delegates of the OIE, Resolution N° 39 was adopted and has established that (http://www.oie.int/fileadmin/Home/eng/About_us/docs/pdf/Session/2017/A_RESO_2017_Public.pdf)

- **The OIE will demonstrate the positive impacts of Public-Private Partnerships (PPPs) in the field of animal health and livestock sector development and disseminate best practices to support OIE Member Countries**

and

- **The Member Countries will use those best practices to promote, develop and implement policies and legislation to incentivise collaborations with the private sector to improve animal health and livestock sector development**

Within this context, the objective of this short survey is to identify PPPs that strengthen Veterinary Services in your country.

The results of this survey will serve as a basis to analyse the role of PPPs, define the required enabling environment and stimulate new PPPs to further strengthen Veterinary Services.

In this questionnaire, a PPP is defined as a collaborative approach in which the public and private sector share resources, responsibilities and risks to achieve common objectives and mutual benefits in a sustainable manner.

PPPs require building formal links between the Veterinary Authority and a number of actors from the private sector, including:

- **private veterinary practitioners;**
- **veterinary para-professionals (VPPs) and community animal health workers;**
- **private companies from the pharmaceutical or food industry;**

- **farmers, producers or trade associations, etc.**

You will receive feedback on the results of this survey after analysis of the data.

Thank you for agreeing to participate in the survey which will take you approximately 20 minutes.

IMPORTANT: please note that if you click on your web browser Back button instead of the survey's Previous button, the responses will not be saved on the current page.
Please make sure to answer all mandatory questions (labelled with *), otherwise you will not be able to move on to the next pages.

3. Introduction à l'enquête

Au cours de la 85ème Session Générale de l'Assemblée Mondiale des Délégués de l'OIE, la Résolution N° 39 a été adoptée et prévoit :(http://www.oie.int/fileadmin/Home/eng/About_us/docs/pdf/Session/2017/A_RESO_2017_Public.pdf)

- **Que l'OIE démontre les impacts positifs des Partenariats Public-Privé (PPP) et diffuse les bonnes pratiques visant à aider les Pays Membres de l'OIE à développer des PPP durables et réussis dans le domaine de la santé animale et du développement du secteur de l'élevage**

et

- **Que les Pays Membres examinent les bonnes pratiques identifiées par l'OIE et promeuvent, développent et mettent en œuvre les politiques et réglementations visant à encourager les collaborations avec le secteur privé en vue d'améliorer la santé animale et le développement du secteur de l'élevage**

Dans ce contexte, l'objectif de cette enquête est d'identifier des PPP qui renforcent les Services Vétérinaires dans votre pays.

Les résultats de l'enquête serviront de base pour analyser le rôle des PPP, définir les facteurs d'environnement qui leur sont favorables, et stimuler la mise en oeuvre de nouveaux PPP, dans l'objectif de renforcer encore les Services Vétérinaires.

Dans ce questionnaire, un Partenariat Public-Privé (PPP) est défini comme une approche collaborative dans laquelle les secteurs public et privé partagent les ressources, les responsabilités et les risques afin d'accomplir des objectifs communs et de réaliser des bénéfices mutuels, et ceci de façon durable.

Les PPP requièrent la constitution de liens formalisés entre l'Autorité Vétérinaire et un certain nombre d'acteurs du secteur privé, incluant :

- **des vétérinaires praticiens;**
- **des para-professionnels vétérinaires et agents communautaires de santé animale**

- **des compagnies privées de secteurs industriels variés tels que les secteurs pharmaceutique ou agro-alimentaire ;**
- **des éleveurs, des associations de producteurs, etc.**

Vous recevrez une synthèse des résultats de cette enquête après analyse des données.

Merci d’avoir accepté de participer à cette enquête qui vous prendra environ 20 minutes.

IMPORTANT: merci de noter qu’en cliquant sur la touche de "retour en arrière" sur votre navigateur Internet au lieu du bouton « Précédent » en fin de page, vos réponses ne seront pas enregistrées.

Merci de bien répondre aux questions obligatoires (labélisées d'un *), sinon vous ne pourrez pas avancer à la page suivante.

WORLD ORGANISATION FOR ANIMAL HEALTH
Protecting animals, preserving our future

Public Private Partnership survey / Enquête Partenariats Public Privé / Encuesta Asociaciones Público-Privadas

4. Introducción de la encuesta

Durante la 85a. Sesión General de la Asamblea Mundial de Delegados de la OIE se adoptó la Resolución No. 39 en la que se ha establecido que (http://www.oie.int/fileadmin/Home/eng/About_us/docs/pdf/Session/2017/A_RESO_2017_Public.pdf):

- **La OIE demostrará los impactos positivos que tienen las asociaciones público-privadas (APP) en el campo de la sanidad animal y el desarrollo del sector pecuario y difundirá las mejores prácticas para apoyar a los Países Miembros,**

y

- **Los Países Miembros utilizarán las mejores prácticas para promover, desarrollar e implementar políticas y leyes que incentiven las colaboraciones con el sector privado para mejorar la sanidad animal y el desarrollo del sector ganadero.**

En este contexto, el objetivo de esta breve encuesta es identificar las APP que apoyan el fortalecimiento de los Servicios Veterinarios de su país.

Los resultados de esta encuesta servirán de base para analizar el rol de las APP, definir el ambiente propicio que se requiere y estimular a nuevas APP para que participen en el fortalecimiento de los Servicios Veterinarios.

Para fines de este cuestionario, una asociación público-privada se define como una estrategia de colaboración en que los sectores público y privado comparten recursos, responsabilidades y riesgos para alcanzar objetivos comunes y beneficios mutuos de forma sustentable.

Las APP requieren de la construcción de vínculos formales entre la Autoridad Veterinaria y los diferentes actores del sector privado, los cuales incluyen:

- **Veterinarios privados;**
- **paraprofesionales de veterinaria (PPV) y personas de la comunidad que trabajan en salud animal;**
- **compañías privadas de la industria farmacéutica y de alimentos;**

- **granjeros, productores o asociaciones de comercio, etc.**

Una vez que los datos sean analizados, usted recibirá una síntesis de los resultados de esta encuesta.

Completar esta encuesta le tomará aproximadamente 20 minutos. Muchas gracias por su participación.

IMPORTANTE: tenga en cuenta que si hace clic en el botón “Ir a la página anterior” de su navegador, en lugar de hacer uso del botón “Anterior” de la presente encuesta, sus respuestas no se guardarán en la página que acaba de completar.

Por favor asegúrese de responder a todas las preguntas obligatorias (marcadas con *), para poder pasar a la siguiente página.

WORLD ORGANISATION FOR ANIMAL HEALTH
Protecting animals, preserving our future

Public Private Partnership survey / Enquête Partenariats Public Privé / Encuesta Asociaciones Público-Privadas

5. Who are you? / Qui êtes-vous? / Información personal

* 2. What is your last name? / Quel est votre nom? / ¿Cuál es su apellido(s)?

* 3. What is your first name? / Quel est votre prénom? / ¿Cuál es su nombre?

* 4. What is your title? / Quel est votre titre? / ¿Cuál es su título?

- Dr.
- Prof.
- Mr.
- Ms.

* 5. Where do you work? / Dans quel secteur travaillez-vous? / ¿Dónde trabaja?

- Public / Public / Sector público
- Private / Privé / Sector privado

Institution's name (please specify) / Nom de votre employeur (merci de préciser) / Nombre de la institución (por favor especifique)

* 6. What is your position? / Quel est votre poste? / ¿Cuál es su cargo dentro de la institución?

* 7. In which country do you work? / Dans quel pays travaillez-vous? / ¿En qué país trabaja?

8. In your country do you have a veterinary statutory body (an autonomous regulatory body for veterinarians and veterinary para-professionals) /
Dans votre pays, y a-t'il un organisme statutaire vétérinaire (un organe autonome de contrôle des vétérinaires et des para-professionnels vétérinaires)? /
¿Existe algún organismo veterinario estatutario en su país (organismo autónomo de control de veterinarios y paraprofesionales de veterinaria)?

- ◯ YES / OUI / SI
- ◯ NO/ NON / NO
- ◯ I don't know / Je ne sais pas / No lo sé

If YES please provide its name / Si OUI, quel est son nom? / Si su respuesta es afirmativa, indique por favor el nombre

* 9. Contact email / Quel est votre email ? / Correo electrónico

* 10. Contact Phone / Quel est votre numéro de téléphone? / Número de teléfono

Please make sure you have answered all the mandatory questions (labelled with *), otherwise you will not be able to move on to the next page /
Merci de bien répondre aux questions obligatoires (labélisées d'un *), sinon vous ne pourrez pas avancer à la page suivante. /
Por favor asegúrese de responder a todas las preguntas obligatorias (marcadas con *), para poder pasar a la siguiente página.

6.

As you know PPPs represent a collaborative approach between the public sector and a wide range of private actors including private companies from the pharmaceutical or food industry; farmer, producer and/or trade associations; private veterinarians; veterinary para-professionals; etc.
Comme vous le savez, les Partenariats Public-Privé (PPP) constituent une approche collaborative entre le secteur public et un panel large d'acteurs privés possibles, incluant des compagnies pharmaceutiques ou agro-alimentaires, des éleveurs, des associations de producteurs, des vétérinaires privés, des para-professionnels vétérinaires; etc.
Las APP representan un esfuerzo de colaboración entre el sector público y un número extenso de actores del sector privado que incluye la industria farmacéutica o de alimentos; granjeros, productores o asociaciones de comercio; veterinarios privados; paraprofesionales de veterinaria; etc.

* 11. Do you have an example of a successful PPP that has or is improving Veterinary Services in your country?
Connaissez-vous un exemple de PPP qui a contribué ou contribue à renforcer les Services Vétérinaires dans votre pays?
¿Cuenta usted con algún ejemplo de una APP exitosa que haya mejorado o que esté mejorando en este momento los Servicios Veterinarios de su país?

- ◯ Yes / Oui / Si
- ◯ No / Non / No

7. PPP success story 1 / PPP cas étude 1 / Historia de éxito de las APP. Caso No. 1

In your country, please can you describe the most successful public-private partnership which results (or resulted) in an improved level or quality of Veterinary Services
Dans votre pays, pouvez-vous décrire le PPP le plus réussi qui a (ou a eu) pour effet l'amélioration du niveau ou de la qualité des Services Vétérinaires ?
Describa la asociación público-privada más exitosa que haya resultado en el mejoramiento del nivel o de la calidad de los Servicios Veterinarios de su país, o que siga aportando a dichas mejoras

* 12. Can you briefly describe the PPP initiative (context, characteristics and any important element of the partnership)
Pouvez-vous brièvement décrire ce PPP (contexte, caractéristiques et tout autre élement important du partenariat)
¿Podría por favor describir brevemente la iniciativa de la asociación público-privada (contexto, características y cualquier elemento importante de la asociación)?

* 13. Current implementation state / Etat actuel du partenariat / Nivel actual de implementación

- ◯ Prospective; In development / Futur; En développement / En proyecto; bajo desarrollo
- ◯ Ongoing / En cours / En curso
- ◯ Past; Ended / Passé; Terminé / Experiencia pasada; finalizada
- ◯ Other (please specify) / Autre (spécifiez) / Otro (por favor especifique)

* 14. Period of implementation / Période de mise en oeuvre / Periodo de implementación

* 15. The collaboration was initiated by: / Cette collaboration a été initiée par: / La colaboración la inició:

- ◯ Private sector / Le secteur privé / El sector privado
- ◯ Public sector / Le secteur public / El sector público
- ◯ True public & private initiative / Initiative conjointe des deux secteurs / Iniciativa de ambos sectores
- ◯ Other (please specify) / Autre (spécifiez) / Otro (por favor especifique)

* 16. Type of interaction between public and private partner(s) (multiple answers allowed)
Type d'interaction entre les partenaires public et privé (plusieurs réponses possibles)
Tipo de interacción entre las partes pública y privada (puede seleccionar varias respuestas)

- ☐ Communication; awareness / Communication; sensibilisation / Comunicación/sensibilización
- ☐ Consultation; facilitation / Consultation; facilitation / Asesoría; facilitación
- ☐ Accreditation; authorization; delegation / Accréditation; habilitation; délégation / Acreditación; autorización; delegación
- ☐ Participation in joint programs / Participation aux programmes d'action communs / Participación en programas conjuntos
- ☐ Other (please specify) / Autre (spécifiez) / Otro (por favor especifique)

* 17. Type of governance mechanism in place? /
Mécanisme de gouvernance mis en place? /
¿Cuál es el tipo de mecanismo de gobernanza establecido?

- ◯ None / Aucun / Ninguno
- ◯ MoU / Protocole d'entente / Memorándum de entendimiento
- ◯ Contract / Contrat / Contrato
- ◯ Legislation / Législation / Ley
- ◯ Sanitary Mandate / Mandat sanitaire / Mandato sanitario
- ◯ Other (please specify) / Autre (spécifiez) / Otro (por favor especifique)

* 18. Public funding mechanism? / Existence d’un financement public? / ¿Tiene financiamiento público?

- Yes / Oui / Si
- No / Non / No

If Yes, please describe the nature of the funding: funding institution (e.g. Government, public donors, public bank,etc...) ; type and amount of resources (e.g. human, material, financial, etc....)

Si oui, pouvez-vous décrire la nature du financement: institution (gouvernement, organisme donateur, banque, etc.), nature et montant des ressources engagées (ressources humaines, matérielles, financières, etc.)

Si su respuesta es afirmativa, describa la naturaleza del financiamiento: la institución financiera (gobierno, donante. banco, ONG, etc.) tipo y cantidad de recursos (recursos humanos, materiales y financieros, etc.)

* 19. Private funding mechanism? / Existence d’un financement privé? / ¿Tiene financiamiento privado?

- Yes / Oui / Si
- No / Non / No

If Yes, please describe the nature of the funding: funding institution (e.g. industries, private donors, private bank,etc...) ; type and amount of resources (e.g. human, material, financial, etc....)

Si oui, pouvez-vous décrire la nature du financement: institution (par ex. entreprises, donateur privé, banque privée, etc.), nature et montant des ressources engagées (ressources humaines, matérielles, financières, etc.)

Si su respuesta es afirmativa, describa la naturaleza del financiamiento: la institución financiera (privado donante, privado banco, etc.) tipo y cantidad de recursos (recursos humanos, materiales y financieros, etc.)

* 20. Type of activities for each party / Type d’activités conduites par chacune des parties / Tipo de actividades que realiza cada sector

Public sector / Secteur public / Sector público

Private sector / Secteur privé / Sector privado

* 21. Name of partners or entities for each party / Nom des partenaires ou entités pour chacune des parties/ Nombre de los socios o entidades de cada uno de los sectores

Public sector / Secteur public / Sector público

Private sector / Secteur privé / Sector privado

* 22. Contact details for each party / Contacts pour chacune des parties / Información de contacto de ambos sectores

Public sector (name, title, email) / Secteur public (nom, titre, email) / Sector público (nombre, título,correo electrónico)

Private sector (name, title, email) / Secteur privé (nom, titre, email) / Sector privado (nombre, título,correo electrónico)

* 23. Please provide details on the animal species targeted by this PPP/ Pouvez-vous préciser les espèces animales ciblées par le PPP ? /
Por favor detalle las especies animales involucradas en esta asociación público-privada

- [ ] all species / toutes espèces / today las especies
- [ ] bovine / bovins / bovina
- [ ] caprine / caprins / caprina
- [ ] ovine / ovins / ovina
- [ ] camelids / camélidés / camelidos
- [ ] avian / aviaire / aves
- [ ] swine / porcins/ cuisons
- [ ] Other (please specify) / Autre (spécifiez) / Otro (por favor especifique)

24. Please specify targeted disease(s) if relevant /
Pouvez-vous préciser la(les) maladie(s) ciblée(s) si cela s’applique /
Por favor especifique las enfermedades involucradas si la información es de relevancia

* 25. Size and scope of impact (descriptive and/or quantitative) / Pouvez-vous décrire l'impact et le quantifier ? / Tamaño y alcance del impacto (descriptivo o cuantitativo)

Number of animals / Nombre d'animaux / Número de animales

Persons involved / Personnes impliquées / Personas involucradas

Impact on health status / Impact sur la santé / Impacto en el estatus sanitario

Others (local employment, economy, etc...) / Autres impacts (économique, emploi local, etc.) / Otros (empleos locales, economía, etc.)

26. How is impact evaluated (if assessed)? /Comment cet impact est-il ou a-t'il été évalué si c'est le cas ? / ¿Cómo se evalúa el impacto (si la evaluación se lleva a cabo)?

* 27. How is this partnership sustainable? What elements have been built into the PPP to ensure its sustainability? /
Quelles mesures sont prises pour assurer la durabilité de cet impact ? Quels éléments ont-ils été inclus dans le PPP pour prendre en compte ces aspects de durabilité ? /
¿Qué medidas se toman para garantizar la sustentabilidad la asociación? ¿Qué elementos se han establecido en la asociación público-privada para asegurar esta sustentabilidad?

* 28. Strengths of the PPP? / Forces du PPP? / ¿Cuáles son las fortalezas de la asociación público-privada?

* 29. Weaknesses of the PPP? / Faiblesses du PPP? / ¿Cuáles son las debilidades de la asociación público-privada?

30. Please provide additional details if needed/ Utilisez cet espace pour nous donner des détails supplémentaires si besoin / Por favor proporcione cualquier otro detalle que considere relevante

WORLD ORGANISATION FOR ANIMAL HEALTH
Protecting animals, preserving our future

Public Private Partnership survey / Enquête Partenariats Public Privé / Encuesta Asociaciones Público-Privadas

8.

As you know PPPs represent collaborative approach between the public sector and a wide range of private actors including private companies from the pharmaceutical or food industry; farmer, producer and/or trade associations; private veterinarians; veterinary para-professionals; etc. / Comme vous le savez, les Partenariats Public-Privé (PPP) constituent une approche collaborative entre le secteur public et un panel large d'acteurs privés possibles, incluant des compagnies pharmaceutiques ou agro-alimentaires, des éleveurs, des associations de producteurs, des vétérinaires privés, des para-professionnels vétérinaires; etc. /
Las APP representan un esfuerzo de colaboración entre el sector público y un número extenso de actores del sector privado que incluye la industria farmacéutica o de alimentos; granjeros, productores o asociaciones de comercio; veterinarios privados; paraprofesionales de veterinaria; etc.

* 31. Do you have another example of a PPP aimed to improve Veterinary Services in your country? / Souhaiteriez-vous partager un autre exemple de PPP qui améliore les Services Vétérinaires dans votre pays ? / ¿Cuenta usted con otro ejemplo en el que una asociación público-privada haya tenido como objetivo mejorar los Servicios Veterinarios de su país?

- ◯ Yes / Oui / Si
- ◯ No / Non / No

Public Private Partnership survey / Enquête Partenariats Public Privé / Encuesta Asociaciones Público-Privadas

9. PPP success story 2 / PPP cas étude 2 / Historia de éxito de las APP. Caso No. 2

Please remember that PPPs represent collaborative approach between the public sector and a wide range of private actors including private companies from the pharmaceutical or food industry; farmer, producer and/or trade associations; private veterinarians; veterinary para-professionals; etc. /

Nous vous rappelons que les PPP représentent une approche collaborative entre le secteur public et un panel large d'acteurs privés possibles, incluant des compagnies pharmaceutiques ou agro-alimentaires, des éleveurs, des associations de producteurs, des vétérinaires privés, des para-professionnels vétérinaires, etc. /

Por favor recuerde que las APP representan un esfuerzo de colaboración entre el sector público y un número extenso de actores del sector privado que incluye la industria farmacéutica o de alimentos; granjeros, productores o asociaciones de comercio; veterinarios privados; paraprofesionales de veterinaria; etc.

* 32. Can you briefly describe the PPP initiative (context, characteristics and any important element of the partnership)
Pouvez-vous brièvement décrire ce PPP (contexte, caractéristiques et tout autre élement important du partenariat)
¿Podría por favor describir brevemente la iniciativa de la asociación público-privada (contexto, características y cualquier elemento importante de la asociación)?

* 33. Current implementation state / Etat actuel du partenariat / Nivel actual de implementación

- ◯ Prospective; In development / Futur; En développement / En proyecto; bajo desarrollo
- ◯ Ongoing / En cours / En curso
- ◯ Past; Ended / Passé; Terminé / Experiencia pasada; finalizada
- ◯ Other (please specify) / Autre (spécifiez) / Otro (por favor especifique)

* 34. Period of implementation / Période de mise en oeuvre / Periodo de implementación

* 35. The collaboration was initiated by: / Cette collaboration a été initiée par: / La colaboración la inició:

- ◯ Private sector / Le secteur privé / El sector privado
- ◯ Public sector / Le secteur public / El sector público
- ◯ True public & private initiative / Initiative conjointe des deux secteurs / Iniciativa de ambos sectores
- ◯ Other (please specify) / Autre (spécifiez) / Otro (por favor especifique)

* 36. Type of interaction between public and private partner(s) (multiple answers allowed)
Type d'interaction entre les partenaires public et privé (plusieurs réponses possibles)
Tipo de interacción entre las partes pública y privada (puede seleccionar varias respuestas)

- ☐ Communication; awareness / Communication; sensibilisation / Comunicación/sensibilización
- ☐ Consultation; facilitation / Consultation; facilitation / Asesoría; facilitación
- ☐ Accreditation; authorization; delegation / Accréditation; habilitation; délégation / Acreditación; autorización; delegación
- ☐ Participation in joint programs / Participation aux programmes d'action communs / Participación en programas conjuntos
- ☐ Other (please specify) / Autre (spécifiez) / Otro (por favor especifique)

* 37. Type of governance mechanism in place? /
Mécanisme de gouvernance mis en place? /
¿Cuál es el tipo de mecanismo de gobernanza establecido?

- ◯ None / Aucun / Ninguno
- ◯ MoU / Protocole d'entente / Memorándum de entendimiento
- ◯ Contract / Contrat / Contrato
- ◯ Legislation / Législation / Ley
- ◯ Sanitary Mandate / Mandat sanitaire / Mandato sanitario
- ◯ Other (please specify) / Autre (spécifiez) / Otro (por favor especifique)

* 38. Public funding mechanism? / Existence d'un financement public? / ¿Tiene financiamiento público?

- ◯ Yes / Oui / Si
- ◯ No / Non / No

If Yes, please describe the nature of the funding: funding institution (e.g. Government, public donors, public bank,etc...) ; type and amount of resources (e.g. human, material, financial, etc....)

Si oui, pouvez-vous décrire la nature du financement: institution (gouvernement, organisme donateur, banque, etc.), nature et montant des ressources engagées (ressources humaines, matérielles, financières, etc.)

Si su respuesta es afirmativa, describa la naturaleza del financiamiento: la institución financiera (gobierno, donante. banco, ONG, etc.) tipo y cantidad de recursos (recursos humanos, materiales y financieros, etc.)

* 39. Private funding mechanism? / Existence d'un financement privé? / ¿Tiene financiamiento privado?

- ◯ Yes / Oui / Si
- ◯ No / Non / No

If Yes, please describe the nature of the funding: funding institution (e.g. industries, private donors, private bank,etc...) ; type and amount of resources (e.g. human, material, financial, etc....)

Si oui, pouvez-vous décrire la nature du financement: institution (par ex. entreprises, donateur privé, banque privée, etc.), nature et montant des ressources engagées (ressources humaines, matérielles, financières, etc.)

Si su respuesta es afirmativa, describa la naturaleza del financiamiento: la institución financiera (privado donante, privado banco, etc.) tipo y cantidad de recursos (recursos humanos, materiales y financieros, etc.)

* 40. Type of activities for each party / Type d'activités conduites par chacune des parties / Tipo de actividades que realiza cada sector

Public sector / Secteur public / Sector público

Private sector / Secteur privé / Sector privado

* 41. Name of partners or entities for each party / Nom des partenaires ou entités pour chacune des parties / Nombre de los socios o entidades de cada uno de los sectores

Public sector / Secteur public / Sector público

Private sector / Secteur privé / Sector privado

* 42. Contact details for each party / Contacts pour chacune des parties / Información de contacto de ambos sectores

Public sector (name, title, email) / Secteur public (nom, titre, email) / Sector público (nombre, título,correo electrónico)

Private sector (name, title, email) / Secteur privé (nom, titre, email) / Sector privado (nombre, título,correo electrónico)

* 43. Please provide details on the animal species targeted by this PPP/ Pouvez-vous préciser les espèces animales ciblées par le PPP ? /
Por favor detalle las especies animales involucradas en esta asociación público-privada

- [ ] all species / toutes espèces / today las especies
- [ ] bovine / bovins / bovina
- [ ] caprine / caprins / caprina
- [ ] ovine / ovins / ovina
- [ ] camelids / camélidés / camelidos
- [ ] avian / aviaire / aves
- [ ] swine / porcins/ cuisons
- [ ] Other (please specify) / Autre (spécifiez) / Otro (por favor especifique)

44. Please specify targeted disease(s) if relevant /
Pouvez-vous préciser la(les) maladie(s) ciblée(s) si cela s'applique /
Por favor especifique las enfermedades involucradas si la información es de relevancia

* 45. Size and scope of impact (descriptive and/or quantitative)/ Pouvez-vous décrire l'impact et le quantifier ? / Tamaño y alcance del impacto (descriptivo o cuantitativo)

Number of animals / Nombre d'animaux / Número de animales

Persons involved / Personnes impliquées / Personas involucradas

Impact on health status / Impact sur la santé / Impacto en el estatus sanitario

Others (local employment, economy, etc...) / Autres impacts (économique, emploi local, etc.) / Otros (empleos locales, economía, etc.)

46. How is impact evaluated (if assessed)? /Comment cet impact est-il ou a-t'il été évalué si c'est le cas ? / ¿Cómo se evalúa el impacto (si la evaluación se lleva a cabo)?

* 47. How is this partnership sustainable? What elements have been built into the PPP to ensure its sustainability? /
Quelles mesures sont prises pour assurer la durabilité de cet impact ? Quels éléments ont-ils été inclus dans le PPP pour prendre en compte ces aspects de durabilité ? /
¿Qué medidas se toman para garantizar la sustentabilidad la asociación? ¿Qué elementos se han establecido en la asociación público-privada para asegurar esta sustentabilidad?

* 48. Strengths of the PPP? / Forces du PPP? / ¿Cuáles son las fortalezas de la asociación público-privada?

* 49. Weaknesses of the PPP? / Faiblesses du PPP? / ¿Cuáles son las debilidades de la asociación público-privada?

50. Please provide additional details if needed/ Utilisez cet espace pour nous donner des détails supplémentaires si besoin / Por favor proporcione cualquier otro detalle que considere relevante

WORLD ORGANISATION FOR ANIMAL HEALTH
Protecting animals, preserving our future

10. PPPs constraints and opportunities / Contraintes et opportunités / Dificultades y oportunidades

* 51. List 2-3 key constraints that impact the existence of PPPs in your country/ Citez 2 ou 3 contraintes principales qui impactent le développement de PPP dans votre pays / Proporcione 2 o 3 aspectos que dificulten la existencia de las APP en su país

Imagine that these challenges were overcome, what kind of PPP would you like to see in your country, to support Veterinary Services /

Dans l'hypothèse où ces contraintes seraient levées, quel type de PPP voudriez-vous voir se mettre en place dans votre pays en appui aux Services Vétérinaires ? /

Imagine que los retos que tienen las APP en su país fueran superados, ¿qué tipo de APP le gustaría ver en su país con el objeto de apoyar los Servicios Veterinarios?

52. Which private partner(s) would be engaged?/ Quel(s) partenaire(s) privé(s) seraient engagé(s)?/ ¿Qué entidades privadas estarían involucradas?

53. Which public partner(s) would be engaged? / Quel(s) partenaire(s) public(s) seraient engagé(s)? / ¿Qué entidades públicas estarían involucradas?

54. What kind of governance mechanisms and types of veterinary services would be provided?/ Quels mécanismes de gouvernance et quels types de services vétérinaires pourraient être mis en place?/ ¿Qué tipo de mecanismos de gobernanza y tipos de servicios veterinarios se proporcionarían?

11. Other comments / Autres commentaires / Otros comentarios

55. Would you like to share any other comments on how PPPs can further improve the efficiency of Veterinary Services /
Souhaitez-vous partager d’autres commentaires sur la façon dont les PPP peuvent contribuer à améliorer les Services Vétérinaires? /
¿Qué otro comentario considera importante sobre cómo las APP pueden seguir mejorando la eficiencia de los Servicios Veterinarios?

56. Other information of interest / Autres informations que vous souhaiteriez partager / Alguna otra información que usted desee compartir

Public Private Partnership survey / Enquête Partenariats Public Privé / Encuesta Asociaciones Público-Privadas

12. Perspectives / Perspectives / Perspectivas

* 57. What would be your expectations regarding feedback from this study?/
Quelles seraient vos attentes sur le retour d'information des résultats de cette étude ?/
¿Que esperaría recibir como resultado de este estudio?

In some regions, we will organise seminars/workshops to facilitate the development of PPPs towards improved Veterinary Services /
Dans plusieurs régions, nous avons prévu d'organiser des séminaires ou des sessions de travail pour faciliter le développement de Partenariats Public-Privé destinés à renforcer les Services Vétérinaires. /
En algunas regiones organizaremos seminarios o talleres para facilitar el desarrollo de APP que mejoren los Servicios Veterinarios.

* 58. Would you be interested to attend such seminars/workshops?/ Seriez-vous intéressés pour participer à ces séminaires? / ¿Estaría interesado en asistir a este tipo de seminarios o talleres?

- Yes / Oui / Si
- No / Non / No

Any Comments / Autres commentaires / Algún comentario adicional

* 59. What would be your expectations on the content of such seminars?/ Quelles seraient vos attentes concernant le contenu de tels séminaires ou sessions de travail? / ¿Cuál esperaría que fuera el contenido de estos seminarios?

Thank you very much for your time and participation!

Please feel free to add any additional comments below

Nous vous remercions d'avoir participé à cette enquête!

N'hésitez pas à partager toute autre remarque ci-dessous

¡Muchas gracias por su tiempo y participación!

Por favor agregue cualquier comentario adicional que considere importante

60. Additional comments / Autres commentaires / Comentarios adicionales
