# Supplementary material for: Typological analysis of public-private partnerships in the veterinary domain
Source: PLoS One. 2019 Oct 31;14(10):e0224079. doi: 10.1371/journal.pone.0224079 (PMC6822735; doi:10.1371/journal.pone.0224079)
Supplement: S2 File — (DOCX) [file pone.0224079.s002.docx]

**Typological analysis of public-private partnerships in the veterinary domain publication : Supporting Information 2.**

### Associations between the four clustering methods

The three methods used in the factorial analysis provided a consistent classification of the PPPs: the three clusters of PPPs were consistently identified by all three methods in cluster 1 (top-left corner), cluster 2 (bottom-left) and cluster 3 (right side) (Figure S1). There was a very good agreement between methods, which brings a high level of confidence in the retained classification.

**Figure S1.** Convergence of the outputs from the 4 factorial analysis methods implemented in this study.

The 3 PPPs (public-private partnerships) clusters are clearly distinguished by the 3 methods: MCA = Multiple Correspondence analysis; MDS = MultiDimensional Scaling; CT = classification Tree, HC = Hierarchical Clustering.

The factorial analysis of data collected through the survey therefore highlighted 3 distinct clusters of PPPs providing the basis for a PPP typology proposal (Figure S2). Two main variables were essential to explain the differences between the 3 clusters: the category of main private partner collaborating with the public sector and the type of interaction between the partners. Other variable categories were identified to further characterize each of the three clusters (Figure S3).

**Figure S2**. Cluster differentiations of the PPPs according to the 3 clustering approaches: the PPPs are grouped in 3 different clusters by the k-means method.

(A); those clusters can be differentiated according to 2 main variables: type of private partners and governance mechanism as shown with the hierarchical clustering and the classification tree methods (B and C)

**Figure S3**. Correlation between variables and MCA dimensions (left side) and relative contribution of the 10 most influential categories (right side).
