# Supplementary material for: Typological analysis of public-private partnerships in the veterinary domain
Source: PLoS One. 2019 Oct 31;14(10):e0224079. doi: 10.1371/journal.pone.0224079 (PMC6822735; doi:10.1371/journal.pone.0224079)
Supplement: S1 Table — (DOCX) [file pone.0224079.s003.docx]

Table S1. List of variables used to charaterize the public-private partnerships

| Type of respondent |
| --- |
| Country name |
| OIE region |
| Country World Bank classification by income 2017 |
| Country PVS collaboration criteria score |
| General objective |
| Specific objective |
| Main modality |
| Main activity |
| Animal species targeted |
| Disease(s) targeted |
| Value chain step |
| Current implementation state |
| Period of implementation |
| Duration |
| Who initated the collaboration |
| Type of public partner |
| Type of private partner |
| International partner |
| International partner type |
| Type of interaction |
| Governance mechanism (description) |
| International partner |
| Type of activity (Public) |
| Type activity (Private) |
| Type of resources |
| Financial resources |
| Public funding mechanism |
| Public funding nature |
| Private funding mechanism |
| Private funding nature |
| Size of impac |
| Type of impact |
| Evaluation of impact |
| Strenghts |
| Weaknesses |
