# Supplementary figures and images for: Typological analysis of public-private partnerships in the veterinary domain
Source: PLoS One. 2019 Oct 31;14(10):e0224079. doi: 10.1371/journal.pone.0224079 (PMC6822735; doi:10.1371/journal.pone.0224079)

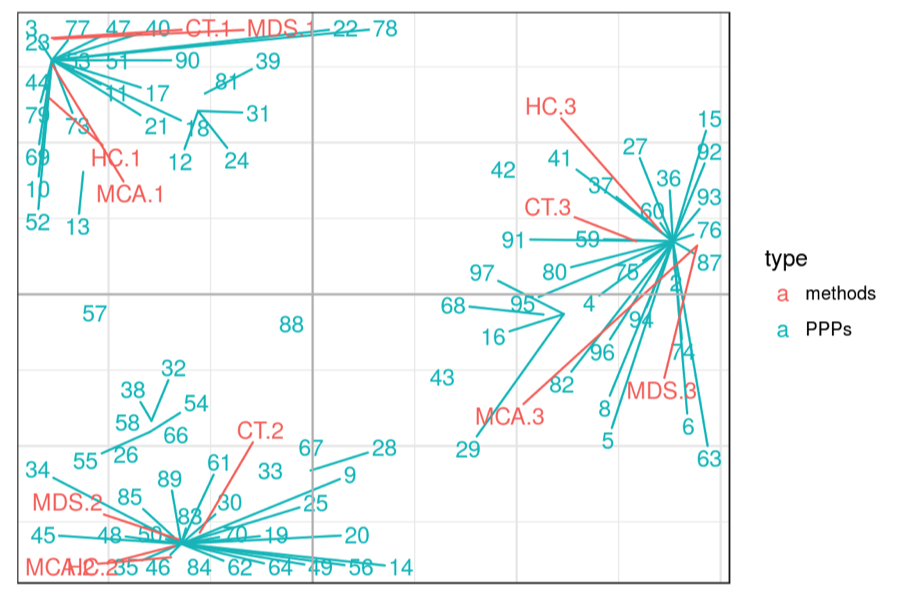

Supplement: S1 Fig — The three PPP (public-private partnership) clusters are clearly distinguished by the three methods: MCA = Multiple Correspondence Analysis; MDS = Multidimensional Scaling; CT = Classification Tree, HC = Hierarchical Clustering. (TIF) [file pone.0224079.s004.tif]

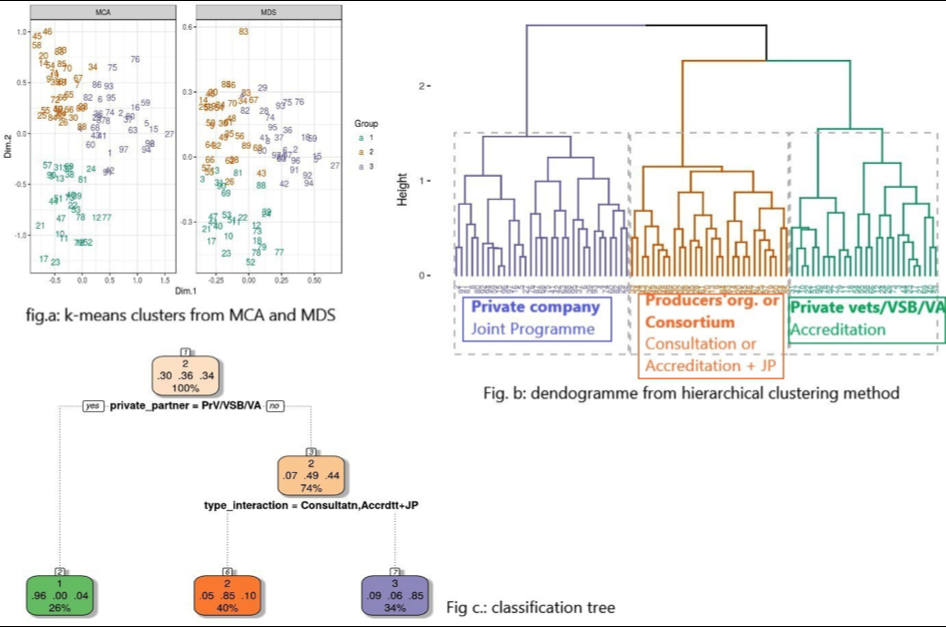

Supplement: S2 Fig — (A); those clusters can be differentiated according to two main variables: the type of private partners and the governance mechanism, as shown with the hierarchical clustering and classification tree methods (B and C). (TIF) [file pone.0224079.s005.tif]

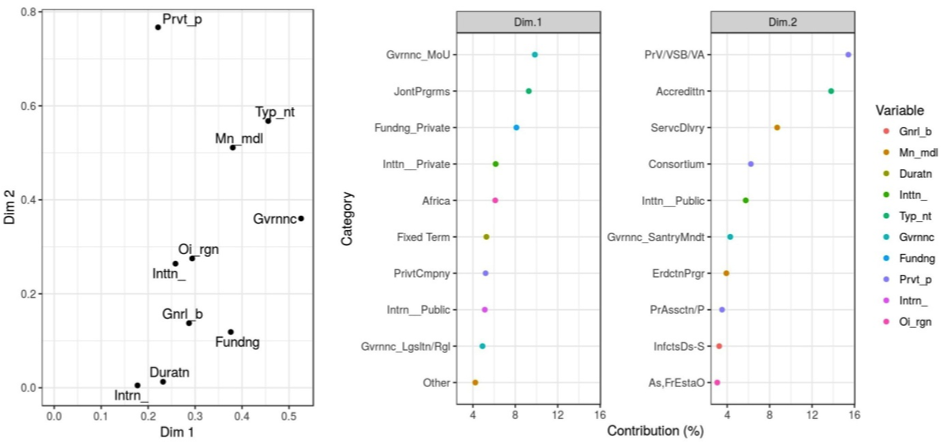

Supplement: S3 Fig — Correlation between variables and MCA dimensions (left side) and relative contribution of the 10 most influential categories (right side). (TIF) [file pone.0224079.s006.tif]
